# The interplay between *miR156/SPL13* and *DFR/WD40–1* regulate drought tolerance in alfalfa

**DOI:** 10.1186/s12870-019-2059-5

**Published:** 2019-10-21

**Authors:** Biruk A. Feyissa, Muhammad Arshad, Margaret Y. Gruber, Susanne E. Kohalmi, Abdelali Hannoufa

**Affiliations:** 1 Agriculture and Agri-Food Canada, 1391 Sandford Street, London, Ontario N5V 4T3 Canada; 2 Department of Biology, University of Western Ontario, 1151 Richmond Street, London, Ontario N6A4B7 Canada; 3 Center of Agricultural Biochemistry and Biotechnology, University of Agriculture, Faisalabad, Pakistan; 4 Agriculture and Agri-Food Canada, 107 Science Place, Saskatoon, Saskatchewan S7N OX2 (retired) Canada

**Keywords:** Alfalfa, Drought, microRNA, miR156, *SQUAMOSA-PROMOTER BINDING PROTEIN-LIKE13*, *WD40–1*

## Abstract

**Background:**

Developing *Medicago sativa* L. (alfalfa) cultivars tolerant to drought is critical for the crop’s sustainable production. miR156 regulates various plant biological functions by silencing SQUAMOSA-PROMOTER BINDING PROTEIN-LIKE (SPL) transcription factors.

**Results:**

To understand the mechanism of miR156-modulated drought stress tolerance in alfalfa we used genotypes with altered expression levels of miR156, miR156-regulated *SPL13*, and *DIHYDROFLAVONOL-4-REDUCTASE* (*DFR*) regulating *WD40–1*. Previously we reported the involvement of miR156 in drought tolerance, but the mechanism and downstream genes involved in this process were not fully studied. Here we illustrate the interplay between miR156/SPL13 and WD40–1/DFR to regulate drought stress by coordinating gene expression with metabolite and physiological strategies. Low to moderate levels of miR156 overexpression suppressed *SPL13* and increased *WD40–1* to fine-tune *DFR* expression for enhanced anthocyanin biosynthesis. This, in combination with other accumulated stress mitigating metabolites and physiological responses, improved drought tolerance. We also demonstrated that SPL13 binds in vivo to the *DFR* promoter to regulate its expression.

**Conclusions:**

Taken together, our results reveal that moderate relative miR156 transcript levels are sufficient to enhance drought resilience in alfalfa by silencing *SPL13* and increasing *WD40–1* expression, whereas higher miR156 overexpression results in drought susceptibility.

## Background

The effects of climate change are expected to result in frequent and extreme weather events causing major damage to crop production [1, 2]. Plants respond to these changes (abiotic stress) by developing different resilience mechanisms at the phenotypic, physiological and molecular levels [3]. To improve plant response, microRNAs provide an opportunity to mend alfalfa traits [4].

MicroRNAs are small RNAs of approximately 16–26 nucleotides in length that regulate gene expression at the posttranscriptional level in a sequence-specific manner [5]. Of the hundreds of microRNAs [6], microRNA156 (miR156) is highly conserved in plants, where it functions by down-regulating a group of SQUAMOSA-PROMOTER BINDING PROTEIN-LIKE (SPL*)* transcription factors [7–9]. There are at least eight members (a to h) of miR156 in *Arabidopsis thaliana*, with g and h unique to this species. A smaller number of miR156 members (a to f) have been discovered in other plant species, including *Medicago truncatula* [10]. SPLs regulate a network of downstream genes affecting plant development and physiology by binding to gene promoters at a consensus DNA sequence NNGTACR (where N = any nucleotide, R = A or G) known as the SPL Binding Domain (SBD) [11–14]. In *Arabidopsis,* miR156 regulates 10 out of 16 *SPL*s, affecting various aspects of plant growth and development [15], whereas in alfalfa, miR156 regulates at least seven *SPL*s (*SPL2,3,4,6,9,12* and *13*) [8]. Despite the conservation of miR156 among plant species, some of its regulation outputs are species-specific [9, 13, 16]. We previously showed that overexpression of miR156 in alfalfa delays flowering time, enhances root nodulation, and improves vegetative and root growth [7, 13]. Many of these traits are associated with abiotic stress tolerance [17, 18]. Moreover, overexpression of miR156d was shown to improve alfalfa’s tolerance to heat [19], salinity [20] and drought stress [21]. miR156-mediated silencing of *SPL2, SPL9* and *SPL11* improved heat, salt and drought stress resilience in *Arabidopsis* and rice [22, 23]. *Arabidopsis* mutants with increased miR156 expression silenced *SPL9,* and enhanced expression of *DIHYDROFLAVONOL-4-REDUCTASE* (*DFR*) and *PRODUCTION OF ANTHOCYANIN PIGMENT 1* (*PAP1*), which resulted in increased anthocyanin accumulation and improved stress tolerance [22]. The enhancement of anthocyanins and proanthocanidins is regulated by transcription factors such as WD40, MYB and bHLH [24, 25]. These secondary metabolites scavenge free radicals during plant abiotic stress [26–28] and function in a coordinated manner with transient stress-related primary metabolites such as proline, galactinol, raffinose and gamma-aminobutyric-acid (GABA) to alleviate stress symptoms [26, 29].

We recently reported that drought stress enhances miR156 expression to improve alfalfa’s resilience to this stress by increasing leaf gas exchange and abscisic acid (ABA), while reducing water loss [21]. Despite these findings, our understanding of how the miR156/SPL network regulates downstream genes such as *DFR* and *WD40–1* to affect stress tolerance in alfalfa is unknown, especially as it relates to drought stress and secondary metabolism. In this study, we investigated the mechanism of how miR156 regulates drought stress response in alfalfa. To that end, we analyzed miR156 over-expressors, *SPL13*-silenced genotypes, *WD40–1* over-expressors and *WD40–*1 RNAi silenced genotypes at the metabolomic, transcriptomic and physiological levels. Moreover, we investigated the binding of SPL13 to the *DFR* promoter to regulate flavonoid biosynthesis. The findings from this report would be useful to understand the mechanisms deployed by miR156 in regulating drought stress and could be used as a tool in marker-assisted breeding to improve alfalfa and potentially other crops.

## Results

### Enhanced miR156 expression improves drought tolerance by altering root architecture and water holding capacity

**Fig. 1:**
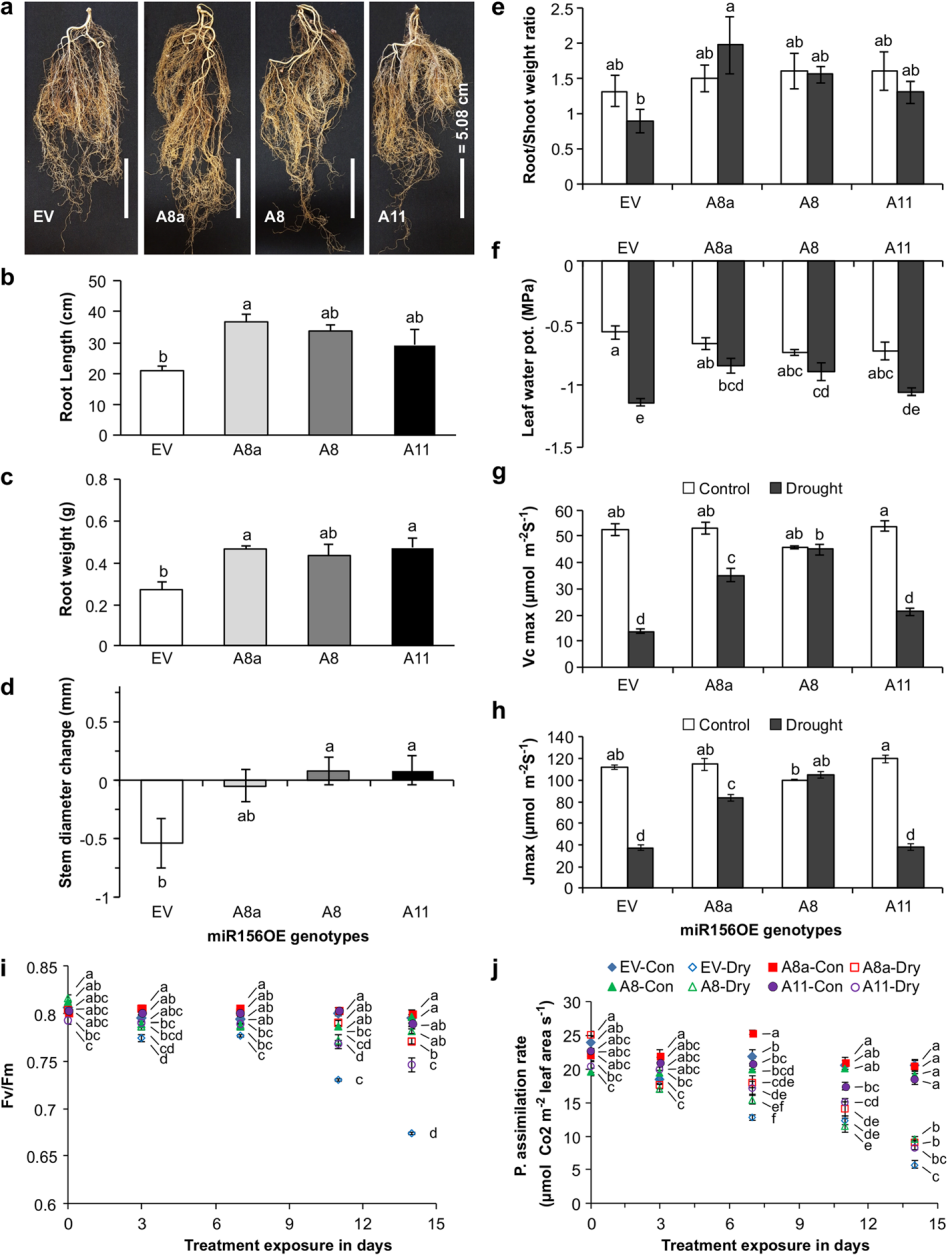
Effects of miR156 overexpression on drought tolerance and physiological responses in alfalfa. **a** Roots of EV and miR156OE plants under drought stress; **b** root length; **c** root weight; **d** stem basal diameter change under drought; **e** root/shoot biomass ratio; **f** leaf water potential; **g** *Vcmax*, maximum rate of rubisco carboxylase activity; **h** *Jmax*, maximum rate of photosynthetic electron transport; **i** dark adapted chlorophyll florescence, Fv/Fm, and **j** photosynthetic assimilation rate in well-watered (control) and drought stressed plants. Values are sample means ± SE, *n* = 4 individual plants except in ‘**d**’, ‘**e**’, ‘**f**’, ‘**i**’, ‘**j**’ where *n* = 5. ANOVA *p* values are provided in Additional file 2: Table S5.1. Significant difference in Post hoc Tukey multiple comparisons test is indicated with different letters. Letters in multiple time point data of ‘**i**’ and ‘**j**’ is analyzed separately

To determine drought stress regulation by miR156, we used one-month-old miR156OE alfalfa plants with low (A8a = 0.5), moderate (A8 = 1.5) and higher (A11 = 2.5) relative miR156 expression levels than the empty vector (EV) [13] grown under drought and well-watered conditions. Root weight, root length, stem basal width and fresh root-to-shoot weight ratios were affected by drought stress depending on the genotype (Fig. 1, Additional file 2: Table S5.1). Relative to EV, A8a had significantly longer roots and increased root biomass (Fig. 1a), with increases of root length up to 1.8-fold (Fig. 1b) and 1.7-fold in root weight (Fig. 1c). The increment of root biomass in A8a was the result of longer roots rather than short and thicker roots (Fig. 1b,c). To understand if the improved root architecture affected plant water potential, we measured leaf water potential [30] and changes in the lower stem diameter before and after drought [31–33]. MiR156OE genotypes, A8a and A8, maintained a higher leaf water potential (Fig. 1f) and also either maintained or increased basal stem diameter (Fig. 1d) while EV plants showed a reduction over the 2 weeks of stress. The unchanged basal stem diameter was accompanied by an increase in root/shoot biomass ratio in A8a and A8 (Fig. 1e).

### miR156 overexpression affects photosynthesis parameters

Since drought stress negatively affects photosynthesis parameters [34], we investigated this effect in miR156OE and EV plants. Accordingly, photosystem II (PS II) chlorophyll fluorescence, Fv/Fm ratio, was measured. Fv/Fm was significantly affected by genotype, drought exposure time, and a combination of both (Additional file 2: Table S5.1). MiR156OE plants maintained higher levels of Fv/Fm ratio (0.75) at later stages (day 11 and 14) comparable to unstressed plants while EV plants showed a gradual reduction to 0.69 after 14 days of drought (Fig. 1i). Furthermore, photosynthesis assimilation rate was significantly affected by genotype and the duration of drought exposure (Additional file 2: Table S5.1). Our data revealed that during drought stress the photosynthetic assimilation rate was higher in A8, gradually decreased in A8a, and further decreased in A11except on day 14 when it was greater than in EV (Fig. 1j).

Moreover, the maximum rate of rubisco carboxylase activity *Vcmax* was maintained at a relatively higher level in A8a and A8 plants while a comparably significant reduction (64–75%) was observed in EV and A11 plants during drought stress (Fig. 1g). In line with this, maximum photosynthetic electron transport rate *Jmax* was also maintained at higher levels in A8a and A8 during drought stress while it was reduced (64%) in EV and A11 plants (Fig. 1h).

### miR156OE plants accumulate anthocyanin and other stress-related secondary metabolites under drought

**Fig. 2:**
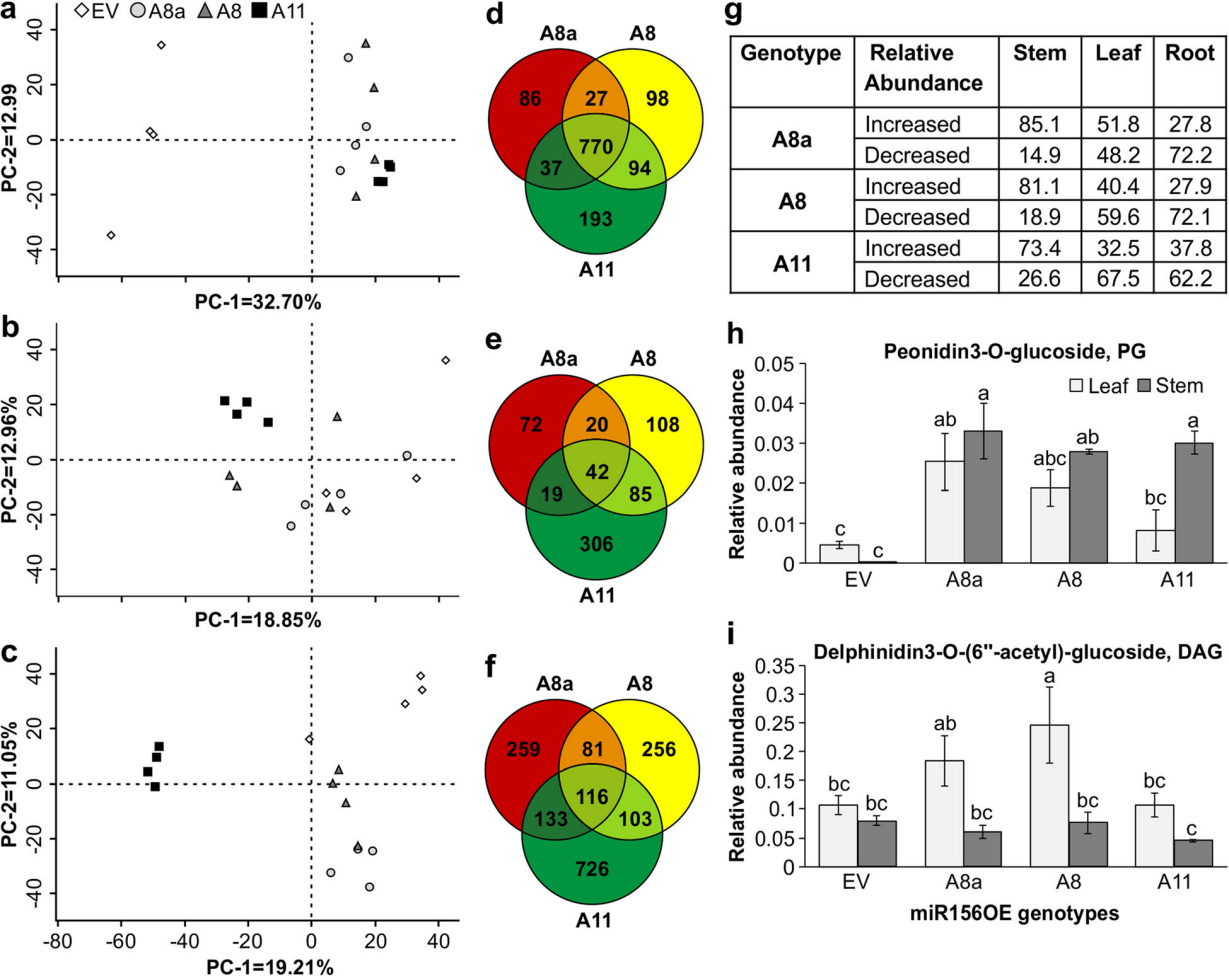
LCMS-based metabolite profiling illustrates distinct profile in miR156OE genotypes during drought stress. **a** Principal component analysis of metabolite profile in stem, **b** leaf, and **c** root tissues under drought stress; **d** metabolite features that are significantly different at *p* < 0.01 from EV plants in tissues of stem, **e** leaf, and **f** root tissues; **g** proportion of metabolite features that are significantly increased (≥ 1.5 log 2 fold change) or decreased (≤ − 1.5 log 2 fold change) relative to EV under drought stress; **h** relative levels of anthocyanin metabolites of peonidin 3-O-glucoside, PG, and **i** delphinidin 3-O-(6″-acetyl)-glucoside, DAG. The relative abundance of metabolites is normalized to an internal standard. Values are sample means ± SE, *n* = 4 individual plants. ANOVA p values are provided in Additional file 2: Table S5. 4. Significant difference in Post hoc Tukey multiple comparisons test is indicated with different letters

Using more than 4000 metabolite features, a Principal Component Analysis (PCA) plot of LCMS-based metabolite profiles depicted a distinct difference between drought-treated EV and miR156OE stem tissues (Fig. 2a). These metabolite features are spectral data generated from metabolites [35, 36]. Principal component-1 (PC-1) contributed 32.7% of the variance and clearly separated EV and miR156OE genotypes stem samples while principal component-2 (PC-2) accounted for 13% of the variance.

Unlike stem tissues (Fig. 2a), roots possessed a differential metabolite features profile for all genotypes with PC-1 and PC-2 variance of 19.21 and 11.05%, respectively (Fig. 2c). On the other hand, leaves of A8a and EV were metabolically closer (Fig. 2b), whereas the higher miR156 expressor, A11, possessed a different metabolic profile, with PC-1 and PC-2 variance of 18.85 and 12.96%, respectively. Based on their significance level and fold change relative to EV, the numbers of metabolite features common or different in stem, leaf and root of miR156OE genotypes under drought stress are presented in Fig. 2d, e and f, respectively. Figure 2d reveals a communal relatively high number of differentially abundant metabolite features (770) between stems of miR156OE and EV plants. The majority (85.1, 81.1, and 73.4% for A8a, A8, and A11, respectively) of the differentially abundant stem metabolites are significantly increased in comparison to EV stem (Fig. 2g). The differential metabolite feature between miR156OE and EV is likely associated with the commonly observed pigmentation of the stem basal internode in miR156OE plants (Additional file 2: Figure S1).

Drought stress induces production of reactive oxygen species (ROS) [37], and plants employ several strategies, including secondary metabolite antioxidants to decrease ROS [38]. Of the many secondary metabolites used by plants as antioxidants, anthocyanins are well documented [39, 40]. Here, levels of anthocyanins such as peonidin 3-O-glucoside (PG) and delphinidin 3-O-(6″-acetyl)-glucoside (DAG) were significantly affected by genotype and tissue (Additional file 2: Table S5.4). LCMS-based metabolite profiling showed anthocyanins and other ROS scavenging phenolic metabolites were increased mainly in stems of low-to-medium miR156 expressors (A8a and A8), although PG was also increased in A11 (Fig. 2h, i and Additional file 2: Table S2). Acylation of the sugar moiety in anthocyanins increases metabolite stability [41, 42]. It remains to be determined whether such acylation is a factor in leaves of A8 having higher levels of DAG relative to A11 and EV resulting in improved drought tolerance (Fig. 2i).

### Alfalfa plants expressing moderate levels of miR156 accumulate stress-related primary metabolites under drought

**Fig. 3:**
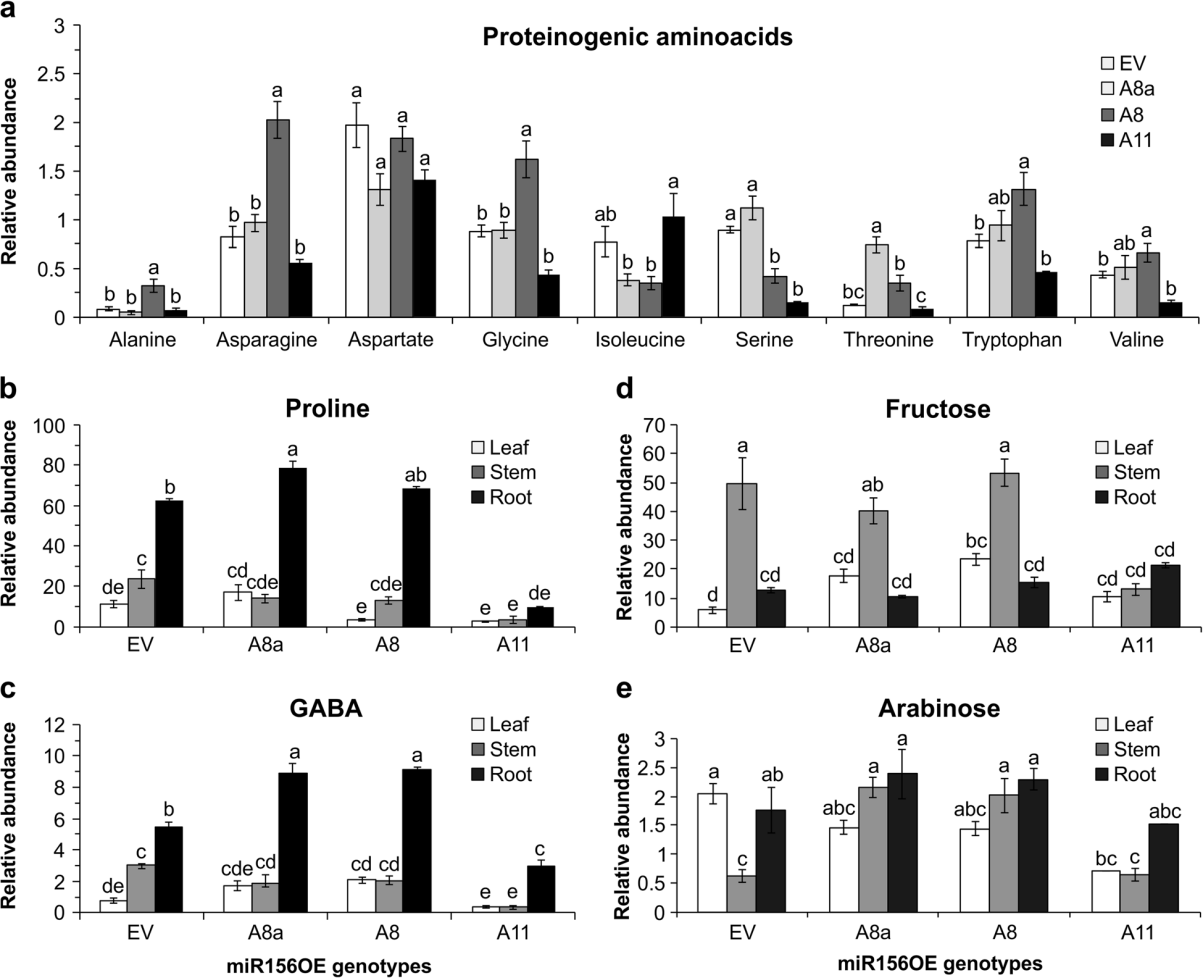
GCMS-based primary metabolite profiling demonstrates drought stress tolerance strategies by miR156. **a** Relative levels of proteinogenic amino acids in leaf tissues during drought stress: alanine, asparagine, aspartate, glycine, isoleucine, serine, threonine, tryptophan and valine; **b** relative levels of metabolites from the γ-aminobutyric acid (GABA) shunt in leaf, stem and root tissues of proline, and **c** GABA; **d** relative levels of sugars from tissues of leaf, stem and root as fructose, and **e** arabinose under drought stress. Values are sample means ± SE, *n* = 4 individual plants. ANOVA p values are provided in Additional file 2: Table S5.5. Significant difference in Post hoc Tukey multiple comparisons test is indicated with different letters

Plants coordinate primary and secondary metabolites for tight metabolite regulation and stress response [27, 28, 43]. Hence, we used GCMS for analysis of primary metabolites to determine their levels during drought stress. Results indicated that metabolite levels were governed by tissue and genotype (Additional file 2: Table S5.5). In general, the relative abundance of proteinogenic amino acids was higher in leaf tissues of moderate miR156OE plants, but reduced in highly overexpressing A11 plants (Fig. 3 and Additional file 2: Table S3). With the exception of valine, which showed no significant differences among stem, root and leaf tissues, levels of proteinogenic amino acids were significantly affected by tissue type and a combination of genotype and tissue (Additional file 2: Table S5.5). Alanine, asparagine, glycine and tryptophan showed a relatively higher abundance in leaves of A8 (Fig. 3a). Interestingly, proline, which functions as an osmolyte to maintain plant water potential [26], was significantly increased in root tissues of A8a, comparable in A8 but was reduced in leaf, stem and root tissues of A11 compared to EV plants (Fig. 3b).

Levels of gamma-aminobutyric acid, GABA, a stress-responsive metabolite that mediates carbon to nitrogen balance between glutamate and succinate in the TCA cycle [29], were enhanced in root tissues of A8 and A8a (Fig. 3c). The higher miR156 over-expressor, A11, on the other hand, reduced GABA levels in all tissues as compared to EV (Fig. 3c).

An increased level of fructose, one of the main sugar sources for the carbon skeleton of downstream metabolites and a source of energy, was observed in leaf tissues of A8 while its levels were unchanged in stems and roots (Fig. 3d). On the other hand, A11 had variable levels of fructose (Fig. 3d), with levels being reduced in stems but comparable in roots.

Conversion of carbon sources from sugars into the downstream pathways including glycolysis and pentose phosphate pathway (PPP) is of great importance in stress response and tolerance [44, 45]. Arabinose, an important component of cell wall polysaccharides, PPP, and a major component of glycoproteins and arabinogalactan proteins, had enhanced levels in stems while it was unchanged in leaf and roots of A8a and A8 while reduced in roots of A11 compared to EV (Fig. 3e and Additional file 2: Table S5.5). A complete list of annotated metabolites using GCMS analysis is presented in Additional file 2: Table S3.

### Overexpression of miR156 affects expression of photosynthesis and flavonoid genes

Our physiological and metabolite profiling analysis showed that alfalfa plants overexpressing miR156 at low-to-moderate levels (A8a and A8) have higher anthocyanin levels (Fig. 2h,i) and maintained higher photosynthetic efficiency during drought stress (Fig. 1g-k). We, therefore, investigated if these are regulated at the molecular level by determining relative transcript levels of genes involved in the flavonoid and photosynthetic pathways. Genotype, tissue and their interaction have a significant impact on the transcript levels of flavonoid biosynthesis *DFR* and *MYB112* genes, although *MYB112* showed little difference between tissues (Additional file 2: Table S5.6). Accordingly, higher transcript levels of *DFR* and *MYB112* were observed in stem and leaf tissues of at least some miR156OE plants.

**Fig. 4:**
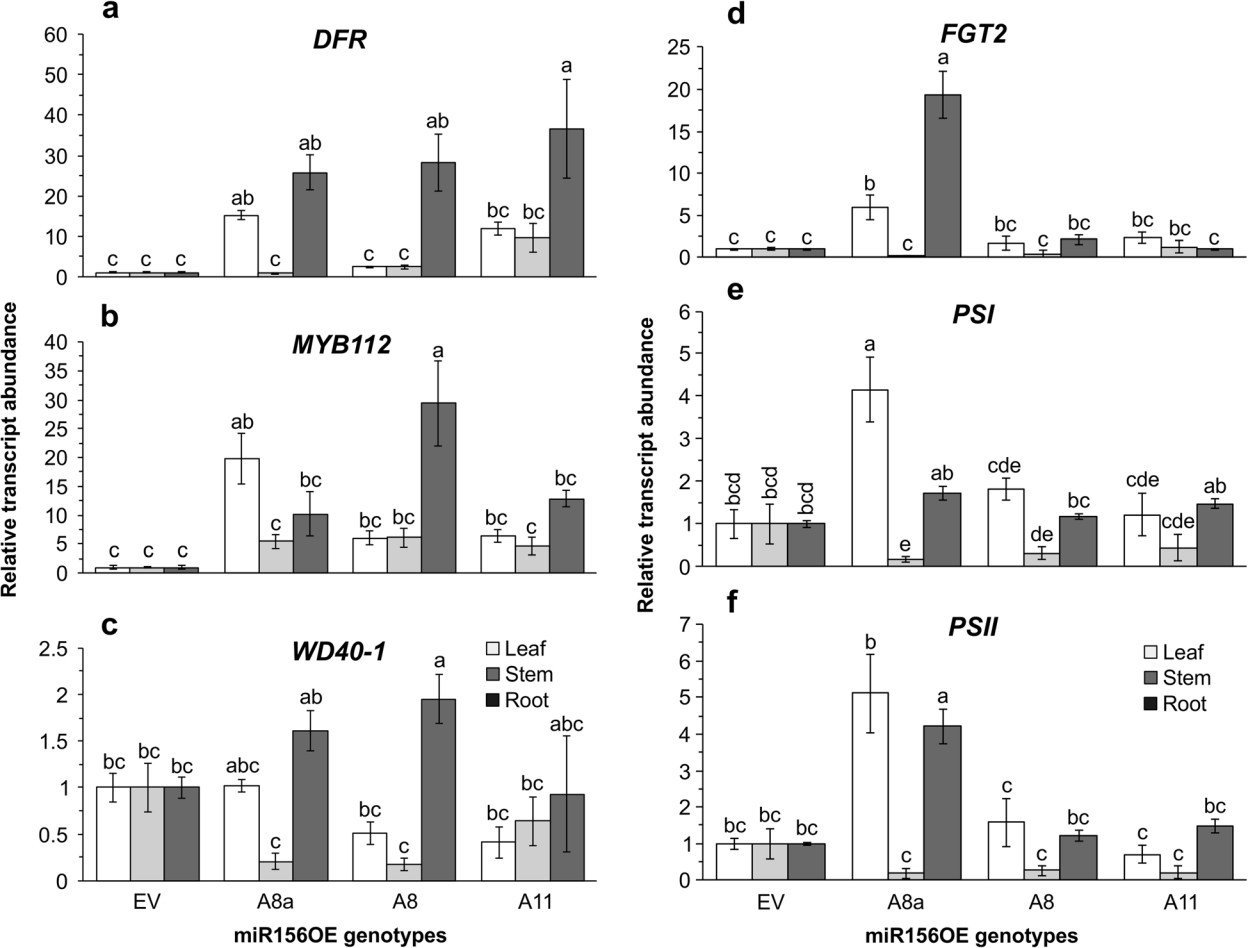
Differential transcript levels of selected genes in the phenylpropanoid pathway and photosystems during drought stress. **a** qRT-PCR based transcript levels of leaf, stem and root tissues of * DIHYDROFLAVONOL-4-REDUCTASE, DFR*; **b** *MYB112*; **c** *WD40–1*; **d** *FLAVONOID GLUCOSYLTRANSFERASE2, FGT2*; **e** *PHOTOSYSTEM I p700 CHLOROPHYLL A APOPROTEIN APS I*, *PSI*; **f** *PHOTOSYSTEM II Q(b)*, *PSII*, *n* = 4 individual plants, values are sample means ± SE. Transcript abundance is relative to empty vector after being normalized to acetyl-CoA carboxylase, *ACC1*, and *ACTIN* housekeeping genes. ANOVA p values are provided in Additional file 2: Table S5.6. Significant difference in Post hoc Tukey multiple comparisons test is indicated with different letters

DFR*,* which catalyses the conversion of dihydroflavonol to leucoanthocyanidin, had two- to 15-fold higher transcription in miR156OE leaf tissues compared to EV (Fig. 4a). *DFR* transcription was also 25 to 35-fold higher in miR156OE root samples. *MYB112* encodes a transcription factor that regulates flavonoid biosynthesis [46]. Its transcript level was five- to 19 times higher in leaf tissues of miR156OE compared to EV while a four-fold higher expression level was observed in miR156OE stem tissues regardless of genotype (Fig. 4b). A slight increment in the expression level of *WD40–1* (1.9-fold), a transcription factor in the phenylpropanoid pathway, was observed in A8 root tissues while it was decreased in stem and leaf tissues (Fig. 4c). Moreover, *FLAVONOID GLUCOSYLTRANSFERASE2* (*FGT2*), which catalyses the transfer of a glycosyl group onto flavonoids, was significantly increased up to six-fold in leaves of A8a while a 19-fold increment was observed in roots (Fig. 4d).

Photosynthesis efficiency related *PHOTOSYSTEM I p700 CHLOROPHYLL A APOPROTEIN APS I* (*PSI*) and *PHOTOSYSTEM II Q(b)* (*PSII*) transcript levels were affected by genotype and tissue type (Additional file 2: Table S5.6). *PSI* and *PSII* transcripts were five and four-fold higher in A8a leaves and roots, respectively (Fig. 4e, f). On the other hand, these two genes were significantly decreased in stems of miR156OE plants (Fig. 4e, f). Stems of miR156OE plants had pigmentation at the basal internode consistent with enhanced anthocyanin accumulation, which interferes with typical green chlorophyll colouration (Additional file 2: Figure S1) [47–49].

### SPL13 regulates physiological responses and anthocyanin accumulation during drought stress in alfalfa

**Fig. 5:**
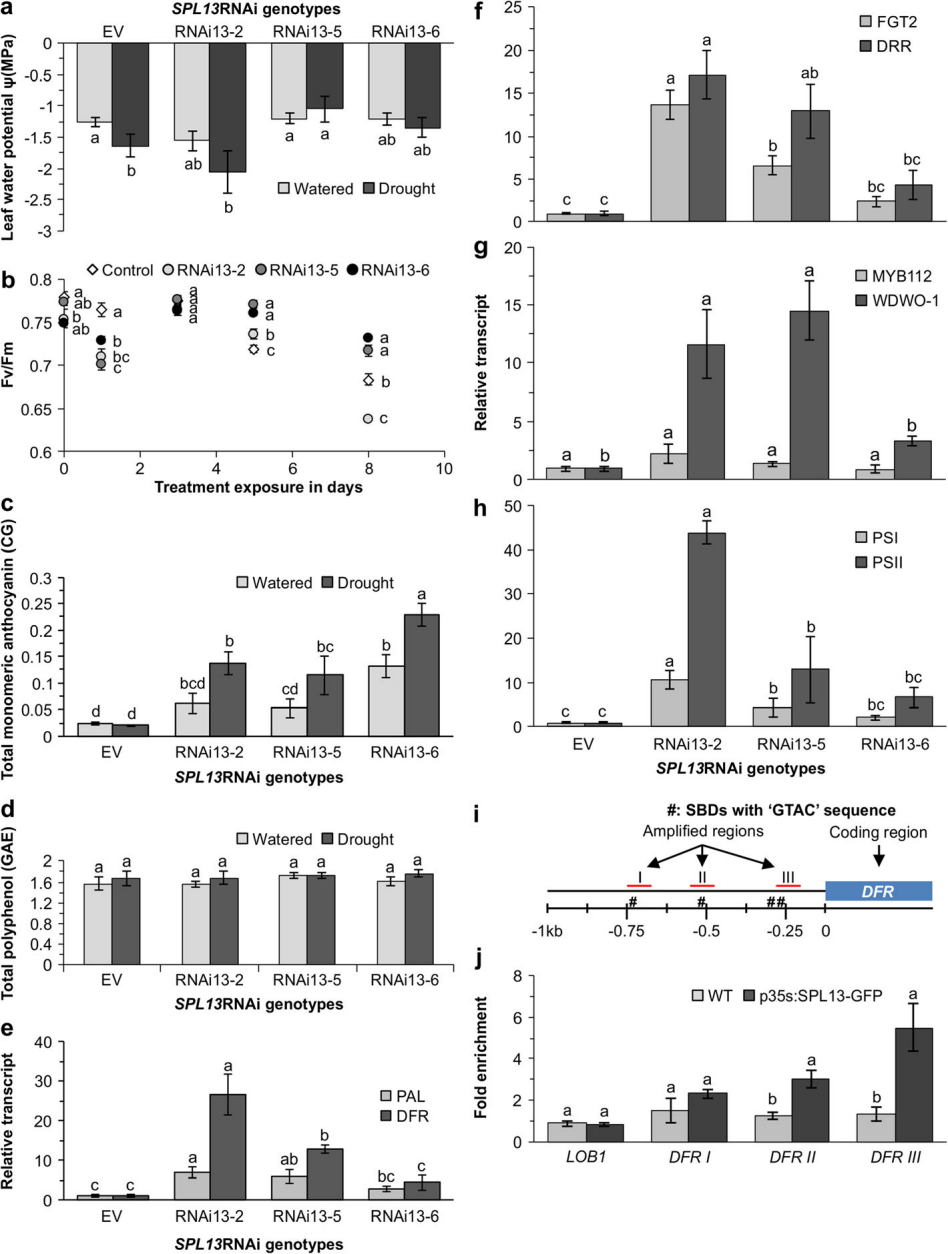
SPL13 silencing regulates drought by coordinated metabolite, transcript, and physiological adjustments. **a** Leaf water potential in *SPL13*RNAi and EV plants; **b** dark adapted chlorophyll florescence, Fv/Fm, during drought stress; **c** total monomeric anthocyanin expressed as cyanidin-o-glucoside equivalent (CG); and **d** total polyphenol content expressed as gallic acid equivalent (GAE); **e** transcript levels of *PHENYLALANINE AMMONIA-LYASE*, *PAL*, and *DIHYDROFLAVONOL-4-REDUCTASE*, *DFR*; **f** *FLAVONOID GLUCOSYLTRANSFERASE2*, FGT2, and *DEHYDRATION RESPONSIVE RD-22-LIKE*, *DRR*; **g** *MYB112* and *WD40–1* transcription factor genes from the phenylpropanoid pathway in stems of *SPL13*RNAi and EV genotypes; **h** transcript levels of *PHOTOSYSTEM I p700 CHLOROPHYLL A APOPROTEIN APS I, PSI*, and *PHOTOSYSTEM II Q(b), PSII* under drought stress; **i** schematic representation of potential SPL13 binding sites in the promoter region of *DFR*, **j** ChIP-qPCR based fold enrichment analysis of SPL13 in p35S:SPL13-GFP and WT plants from means of n = three individual plants where *LATERAL ORGAN BOUNDARES-1, LOB1,* is used as a negative control. Values are means ± SE, light gray bars in ‘**a**’, ‘**c**’ and ‘**d**’ represent values under well-watered condition while dark gray bars represent values under drought stressed conditions. Relative transcript levels in ‘**e**’, ‘**f’, ‘g’** and ‘**h’** are shown relative to EV after being normalized to acetyl-CoA carboxylase, *ACC1*, and *ACTIN* housekeeping genes. ANOVA p values are provided in Additional file 2: Table S5.2, S5.7 and S5.8. Significant difference in Post hoc Tukey multiple comparisons test is indicated with different letters. Letters in multiple time point data of ‘**b**’ is analyzed separately

Since miR156 functions in alfalfa by downregulating *SPL* genes, including *SPL13* [8, 13], we investigated the effect of drought on the physiological and phenotypic parameters of alfalfa plants having RNAi-silenced *SPL13.* We previously reported that a green, normal appearing phenotype accompanied enhanced root development in *SPL13*RNAi genotypes under drought [21]. In the current study, leaf water potential was significantly affected by genotype under drought stress (Additional file 2: Table S5.2). In line with this, *SPL13*RNAi-5 and *SPL13*RNAi-6 plants maintained higher midday leaf water potential during drought stress (Fig. 5a). Moreover, photosynthesis efficiency parameters showed that *SPL13*RNAi-5 and *SPL13*RNAi-6 with moderate *SPL13* silencing [21] maintained a higher Fv/Fm ratio of 0.74 (Fig. 5b) after 8 days of drought stress. The level of Fv/Fm is significantly affected by genotype, length of drought exposure and a combination of both (Additional file 2: Table S5.2). As a stress tolerance strategy, plants use flavonoids such as anthocyanin to scavenge ROS, and in our study we observed that *SPL13*RNAi-6 plants had a significantly higher basal monomeric anthocyanin level under a well-watered condition (Fig. 5c). Interestingly, all *SPL13*RNAi genotypes accumulated a higher level of total monomeric anthocyanin during drought stress while levels in EV did not change (Fig. 5c). A comparable total polyphenol content was mainatined by all genotypes regardless of whether the plants were under well-watered or drought conditions (Fig. 5d).

### Flavonoid- and photosynthesis-related genes are enhanced in *SPL13*-silenced plants

To understand whether the observed increase in the level of total monomeric anthocyanin and maintenance of photosynthesis efficiency under drought stress is regulated at the transcript level we analysed the expression levels of anthocyanin-related and dehydration responsive genes. Our results showed that there were significant differences between genotypes under drought and control conditions (Fig. 5e-h and Additional file 2: Table S5.7). As expected, the transcript level of *PHENYLALANINE AMMONIA-LYASE*, *PAL,* the first committed step in the phenylpropanoid pathway, was significantly higher in two out of three *SPL13*RNAi genotypes (Fig. 5e). Similarly, *DFR* and *FGT2* were also higher in two out of three *SPL13*RNAi genotypes (Fig. 5e,f). These consistently higher levels of *PAL*, *DFR* and *FGT2* transcripts suggest that the induction of flavonoid biosynthesis in response to drought stress is regulated by *SPL13*. In addition, the *DEHYDRATION RESPONSIVE RD-22-LIKE* (*DRR*) gene, which is regulated by MYB and MYC transcription factors and induced by drought and ABA [50, 51], was also expressed four- to 17-fold higher in *SPL13*RNAi plants (Fig. 5f). In line with that, the transcription factor *WD40–1* was increased three- to 14-fold in *SPL13RNAi* plants during drought stress (Fig. 5g). For photosynthesis-related genes, we analysed the transcript levels of *PSI* and *PSII* and found a two- to 10-fold and six to 43-fold increase in expression levels, respectively, in *SPL13*RNAi plants relative to EV (Fig. 5h), consistent with results in A8a and A8 genotypes (Fig. 4e, f).

### SPL13 is a direct regulator of *DFR*

miR156 regulates the expression level of *SPL*s including *SPL13* in alfalfa [8]. Given that *DFR* has four putative SBD binding motifs with core GTAC sequence in the promoter region (Fig. 5i and Additional file 2: Figure S3), we studied the occupancy of SPL13 in the promoter region of *DFR* using ChIP-qPCR in p35S:SPL13-GFP plants. The transgenic (p35S:SPL13-GFP) alfalfa plants were developed previously by our group [52]. We selected three regions (I, II & III) with the conserved SBD core sequences located at 750, 544 and 260 bp, respectively, upstream of the translation start codon of *DFR* as potential SPL13 binding sites, and we tested them for SPL13 occupancy. *LATERAL ORGAN BOUNDARIES-LIKE1*, *LOB1,* was used as a negative control for ChIP-qPCR due to the low SPL13 binding ability to this gene despite the presence of a putative SBD sequence [52]. Compared to WT, p35S:SPL13-GFP plants were significantly higher in SPL13 binding to the *DFR* promoter region (Fig. 5j and Additional file 2: Table S5.8). There is a preferential binding of SPL13 towards the two most downstream putative SBD regions (II & III) in the *DFR* promoter while region I did not show strong binding (Fig. 5i, j and Additional file 2: Figure S3). Of the three regions, region III showed the strongest binding to SPL13 (Fig. 5i, j), indicating that SPL13 could bind directly to *DFR* to regulate its expression.

### WD40–1 positively regulates *DFR* expression and drought tolerance

With the observed higher expression level of *WD40–1* and flavonoid accumulation in miR156OE genotypes during drought stress and a finding from literature regarding the involvment of WD40–1 in the phenylpropanoid pathway [53], we aimed to investigate whether miR156 or SPL13 directly regulate the expression of *WD40–1*. Hence, we investigated the presence of conserved SPL binding (SBD) motifs in the promoter region of *WD40–1*. We used genome walking (GenomeWalker Clonetech Laboratories, Inc.) to obtain the promoter region sequence of *WD40–1*. However, we could not find either a miR156 target sequence or a SBD motif and thus concluded an indirect regulation of *WD40–1* by miR156 or SPL13 (Additional file 2: Figure S4).

**Fig. 6:**
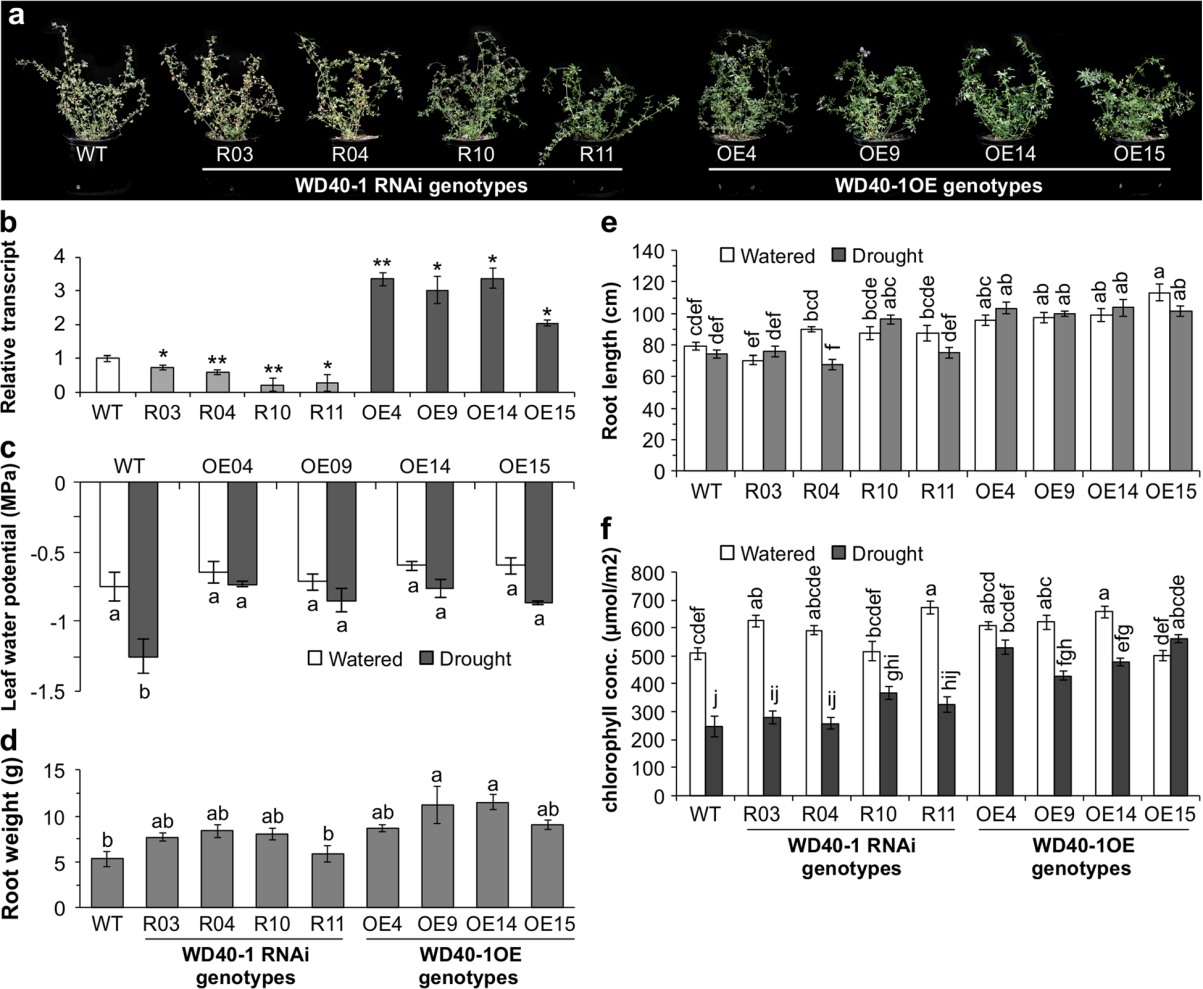
WD40–1 enhances drought tolerance in alfalfa. **a** above ground phenotypes of WT, four *WD40–1R*NAi and four WD40–1OE genotypes during drought stress; **b** transcript levels of *WD40–1* in WT, *WD40–1*RNAi WD40–1OE genotypes used for the study; **c** leaf water potential in WT and WD40–1OE genotypes under well-watered and drought stress condition; **d** root weight in drought stressed WT, *WD40–1*RNAi and WD40–1OE plants; **e** root length in well-watered and drought stressed WT, *WD40–1*RNAi and WD40–1OE plants; and **f** chlorophyll concentration in well-watered and drought stressed WT, *WD40–1*RNAi and WD40–1OE plants. Values are means ± SE; *n* = 4 individual plants for ‘**b**’ to ‘**e**’ while *n* = 20 in ‘**f**’. ANOVA p values are provided in Additional file 2: Table S5.3. Significant difference in Post hoc Tukey multiple comparisons test is indicated with different letters

To further understand the potential role of WD40–1 in alfalfa drought tolerance, we generated plants with overexpressed (OE) or silenced (RNAi) *WD40–1* and exposed these plants to drought stress. We used four different event-derived plants of WD40–1OE (OE04, OE09, OE14 and OE15) and *WD40–1*RNAi (RNAi03, RNAi04, RNAi10 and RNAi11) in comparison to WT plants (Fig. 6a, b). *WD40–1* overexpressing genotypes were drought tolerant while the RNAi silenced *WD40–1* genotypes were susceptible to drought stress (Fig. 6a, Additional file 2: Table S5.3). We investigated phenotypic and physiological responses such as root development, cholorophyll concentration and leaf water potential during drought stress and well-watered conditions.

WD40–1OE genotypes maintained a higher leaf water potential during drought stress (Fig. 6c) as compared to WT and *WD40–1*RNAi genotypes (data not shown). WD40–1OE genotypes developed longer roots and associated root weight (Fig. 6d, e, Additional file 2: Table S5.3). Moreover, WD40–1OE genotypes also maintained higher level of leaf chlorophyll concentration during drought stress (Fig. 6f, Additional file 2: Table S5.3).

**Fig. 7:**
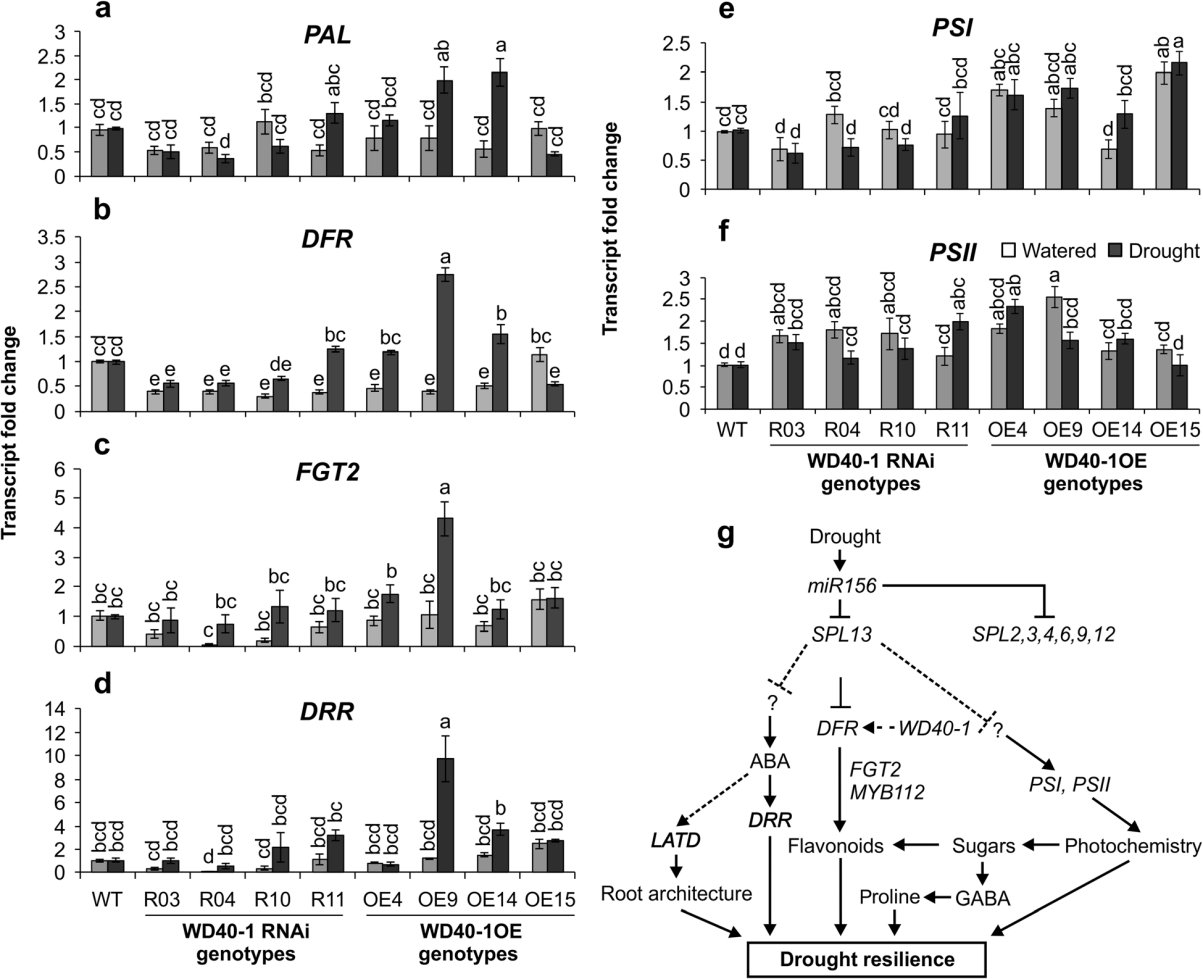
*WD40–1* regulates transcript levels of genes in the phenylpropanoid pathway and photosystem during drought stress. **a** Transcript levels of *PHENYLALANINE AMMONIA-LYASE***,** *PAL*; **b** *DIHYDROFLAVONOL-4-REDUCTASE*, *DFR*; **c** *FLAVONOID GLUCOSYLTRANSFERASE2*, *FGT2*; **d** *DEHYDRATION RESPONSIVE RD-22-LIKE*, *DRR*; **(e)** *PHOTOSYSTEM I p700 CHLOROPHYLL A APOPROTEIN APS I, PSI*; **f** *PHOTOSYSTEM II Q(b), PSII*. Transcript levels are shown relative to EV after being normalized to acetyl-CoA carboxylase, *ACC1*, and *ACTIN* housekeeping. Values are means ± SE, n = 4 individual plants, ANOVA p values are provided in Additional file 2: Table S5.9; **g** schematic representation of miR156-based alfalfa drought resilience model system**.** Solid line represents an experimentally confirmed mechanism while broken lines are hypothesized functions. Arrow heads indicate positive regulation while line heads indicate negative regulation. Significant difference in Post hoc Tukey multiple comparisons test is indicated with different letters

To understand the role of WD40–1 in regulating drought stress through possible interaction with *DFR* and other genes in the phenylpropanoid/flavonoid pathway [24], we measured transcript levels of phenylpropanoid-assosciated genes under drought and well-watered conditions in *WD40–1* silenced and over-expressing genotypes. Accordingly, an increase in *WD40–1* expression enhanced *DFR*, *PAL* and *FGT2* transcripts during drought stress while levels similar to that of WT were observed when plants were kept under well-watered condition (Fig. 7a, b, c, Additional file 2: Table S5.8). Moreover, the ABA-related dehydration responsive gene, *DRR*, and photosynthesis related genes, *PSI* and *PSII*, were increased in WD40–1OE genotypes compared to *WD40–1*RNAi and WT plants (Fig. 7d, e, f, Additional file 2: Table S5.8).

## Discussion

Drought is one of the main factors that impair plant growth and development [54]. Plants respond to drought by showing deleterious effects, or by engaging in adaptive responses involving various molecular, biochemical and physiological strategies [55–57]. In this study, we used miR156OE, *WD40–1*OE, *WD40–1*RNAi, *SPL13*RNAi and GFP-tagged SPL13 genotypes to investigate the molecular and physiological strategies used by miR156 to regulate drought stress in alfalfa.

### Moderate levels of miR156 overexpression, *WD40–1* overexpression or *SPL13* silencing are sufficient to induce phenotypic and physiological drought tolerance strategies in alfalfa

Of the different plant organs that respond to soil water deficit, roots are first to encounter changes in the rhizosphere. Findings in model plants showed initiation and elongation of lateral roots in drought tolerant genotypes to improve water uptake [58, 59]. In this study, we observed a significant increase in root length accompanied by higher root biomass in plants moderately over-expressing miR156 (A8a and A8) and *WD40–1*. This is associated with a reportedly enhanced level of ABA [21] in miR156 overexpressing genotypes under drought stress. ABA enhances primary and lateral root development by regulating the expression of *LATERAL ROOT ORGAN DEFECTIVE* (*LATD*) gene [60]. Moreover, miR156 contributes to root development by silencing *AtSPL10* to decrease the expression of *AGAMOUS-LIKE MADS box protein 79* (*AGL79)* in Arabidopsis [61]. Accordingly, the enhanced root development under drought stress helps alfalfa plants to better access water from deeper soil surface. This finding is consistent with our previous report that showed increased root length in miR156OE and *SPL13*RNAi genotypes under drought conditions [21]. Moreover, moderate miR156OE, *SPL13*RNAi and WD40–1OE genotypes had higher leaf water potential despite their exposure to drought conditions. The observed drought tolerance in miR156OE (A8a and A8), WD40–1OE and *SPL13*RNAi genotypes suggests this trait is at least partially negatively regulated by SPL13 and positively by miR156 and *WD40–1*.

Photosynthesis is negatively impacted by drought stress in alfalfa and other plant species [34, 62]. Of the many photosynthesis efficiency parameters, Fv/Fm reflects the maximum quantum efficiency of PSII photochemistry possible in a dark-adapted state, and is considered a good stress indicator in plants [63–67]. Therefore, maintaining a higher Fv/Fm was one of the parameters used in selecting abiotic stress tolerant cultivars of tomato and wheat [64, 68, 69]. The observed higher level of Fv/Fm in A8a and A8 genotypes in the current study suggests that their leaves may have a functional photosynthetic unit, in agreement with the observed maintained photosynthesis assimilation rate under drought. The observed higher *Vcmax* (Rubisco carboxylase activity) and *Jmax* (electron transport rate) in A8a and A8 under drought further illustrate the maintenance of their photosystem despite drought stress. Such physiological adjustments were low to absent in A11 plants which showed susceptibility to drought stress. We also observed a higher Fv/Fm ratio in *SPL13*RNAi-5 and *SPL13*RNAi-6, which is consistent with our previously reported finding of increased photosynthetic assimilation rate in drought-treated *SPL13*RNAi genotypes [21]. This suggests that the maintenance of a higher photosynthetic assimilation rate, *Vcmax*, *Jmax* and high Fv/Fm ratio during drought stress in miR156OE and WD40–1OE genotypes may be regulated at least in part by SPL13 and *WD40–1*.

### miR156 overexpression enhances accumulation of stress-related metabolites

The impact of environmental perturbations on plant metabolism varies among plant species, cultivars, and tissues considered [70]. Accumulation of specific secondary and transient primary metabolites (primary metabolites that are direct precursors of secondary metabolites) in various tissues are used in part to mitigate drought stress [27, 28, 71, 72]. Naya et al. [73] indicated the role of carbon metabolism and oxidative damage on nitrogenase activity reduction during moderate and higher drought stress levels in alfalfa. Other studies in *M. truncatula*, have shown a decrease in symbiotic nitrogen fixation under drought stress resulting in low levels of nitrogen-based metabolites [74].

In our study, alfalfa with a moderately enhanced expression of miR156 caused accumulation of anthocyanins, flavonols, and proteinogenic amino acids in leaf and stem tissues. The accumulation of these metabolites may help the plant to scavenge ROS produced during drought stress [40]. Moreover, these metabolites could help the plants to reduce water loss, and further absorb any remaining tightly bound water from the soil by lowering the osmotic balance in the root tissues. The high level of GABA in leaf, stem and root tissues of A8a and A8 should maintain a carbon-to-nitrogen balance through a GABA shunt bypassing the decarboxylation part of the TCA cycle [29]. The importance of GABA in mediating abiotic stress has been well documented in various plant species, including *Arabidopsis* [75], black pepper [76] and bentgrass [77]. Proline was also increased in A8a and A8 but not in A11 roots to regulate osmotic homeostasis as reported in other studies [21, 26]. The relatively lower level of proline abundance in roots of the highest miR156 overexpressor, A11, might have prevented these plants from maintaining high water levels in their system (Fig. 1g). The higher level of fructose and arabinose in leaf and stem tissues of drought-treated moderate miR156 expressors respectively could provide an energy source and/or an osmolyte. The higher sugar level suggests an actively functioning photosynthetic assimilation with the potential to supplement a carbon source for downstream metabolites. This is consistent with a previous finding that drought-stressed alfalfa plants accumulate sugars [78]. Moreover, the increased total monomeric anthocyanin and comparable total polyphenol levels in *SPL13*RNAi genotypes illustrated a targeted enhancement of flavonoids at least partially governed by silencing SPL13 in alfalfa to scavenge ROS during drought stress.

### miR156, WD40–1 and SPL13 regulate phenylpropanoid and photosystem genes under drought

Due to the various roles that polyphenols play in stress response, efforts have been made to increase their levels in many plants, including alfalfa [79]. Enhanced accumulation of flavonoids and proanthocyanidins (PA) in alfalfa has important quality implications for animal feed, as moderate amounts of PA tend to reduce bloating in ruminant animals [80–82]. In our study, we found that phenylpropanoid pathway-related genes are enhanced in moderately overexpressing miR156 alfalfa plants, which is consistent with the increase in anthocyanin and flavonol levels in these plants. *DFR, WD40–1* and *MYB112* were higher in A8a and A8 during drought, contributing to anthocyanin accumulation. Similarly, *SPL13*RNAi genotypes showed enhanced levels of *DFR, FGT2* and *PAL* transcripts associated with enhanced level of total monomeric anthocyanin, indicating enhancement of the phenylpropanoid/flavonoid pathway. In another study, *Arabidopsis* plants overexpressing miR156 accumulated anthocyanin in response to salt and mannitol (mimicking drought) treatments by increasing *DFR* expression [23]. The enhanced *DFR* expression level in *Arabidopsis* was regulated by silencing *SPL9* [23]. Our findings suggest that accumulation of anthocyanins and other polyphenols may be regulated via SPL13 in alfalfa. Moreover, the enhanced level of *DFR* in WD40–1OE plants and reduced in *WD40–1*RNAi plants suggests that *DFR* is positively regulated by the WD40–1 to promote flavonoid biosynthesis, but the mechanism of this regulation remains to be investigated.

To investigate whether the higher photosynthetic assimilation rate during drought stress in *SPL13*RNAi [21] and also WD40–1OE, *WD40–1*RNAi and miR156OE genotypes (current study) are regulated at the transcriptional level, we investigated expression of genes mediating photosynthesis. We found that *PSI* and *PSII* were significantly increased in moderately overexpressing miR156OE genotypes and *SPL13*RNAi genotypes upon drought. Previously, we reported an increased abundance of ABA, which regulates stomatal aperture by active hydrolysis during drought stress in miR156OE A8 plants [21]. In the current study, we examined expression of the ABA-induced dehydration responsive gene (*RD22*) and found it to be significantly increased in *SPL13*RNAi plants during drought stress. The consistent observation of higher polyphenols and photosystem assimilation rate with associated transcripts during drought stress in moderate miR156OE and *SPL13*RNAi genotypes suggests a drought regulation strategy of miR156.

### SPL13 binds to *DFR* to regulate its expression and flavonoid biosynthesis

To investigate whether the increased flavonoid accumulation and expression of phenylpropanoid-associated genes, especially *DFR*, are directly regulated by the miR156-SPL13 module, we conducted a ChIP-qPCR analysis to determine binding of SPL13 to *DFR*. DFR catalyses flavonoid biosynthesis by reducing dihydroflavonols to leucoanthocyanidins playing a critical role in anthocyanin biosynthesis [83]. A previous report showed SPL9 directly regulates the expression level of *DFR* to enhance accumulation of anthocyanin in response to NaCl and mannitol treatment in *Arabidopsis* [23]. In the current study, we showed increased *DFR* expression during drought stress in moderately overexpressing miR156 and *SPL13*RNAi plants. Accordingly, we selected *DFR* to test for SPL13 binding, given the presence of multiple potential SBD core GTAC sequences in the *DFR* promoter. The fold enrichment from ChIP-qPCR showed the strongest SPL13 binding was observed at region III of the *DFR* promoter, which is located closest (260 bp) to the *DFR* coding sequence. This is in line with reports that showed the conserved core SBD element is not by itself sufficient for SPL binding, but also determined by the position of the SBD and the flanking DNA sequences [11, 84, 85]. SPL13 acts as a transcriptional suppressor of *DFR* during drought stress as confirmed by higher expression of *DFR* in *SPL13*RNAi and miR156OE plants.

## Conclusions

We recently reported that miR156 regulates drought tolerance in alfalfa by silencing *SPL13* [21]. Understanding the mechanisms deployed by miR156 in drought tolerance could be exploited as a tool in crops for marker-assisted breeding. In the current study, we investigated metabolomic, physiological and molecular mechanisms to show how low- to moderate levels of miR156 expression is sufficient to induce drought tolerance in alfalfa. Moderate levels of miR156 in genotypes of A8a and A8 induced accumulation of stress mitigating metabolites, such as anthocyanins, flavonols, GABA, proline and others in the leaf, stem and root tissues. These metabolites could help the plants to scavenge ROS, reduce water loss and further absorb any remaining tightly bound water from the soil by lowering the osmotic balance in the root tissues. In addition, the plants showed physiological adjustments such as improved photosynthetic assimilation rate, maintained Fv/Fm ratio, and enhanced root growth and development. The relatively low levels of stress mitigating metabolites and reduced physiological adjustments may have resulted in drought susceptibility in the highest miR156 overexpressor (A11). We also determined direct binding of *SPL13* to the *DFR* promoter*. SPL13* acts as a transcriptional suppressor of *DFR* during drought stress as confirmed by higher expression of *DFR* in *SPL13*RNAi and miR156OE plants. Similar observation of SPLs suppressing the expression of *DFR* has been reported in *Arabidopsis* [86] where *SPL9* silenced *DFR* in response to salt and mannitol treatment [23]. Moreover, we detected an increase in expression of genes involved in the phenylpropanoid and photosynthetic pathways, including *DFR, MYB112, PSI* and *PSII* in miR156OE plants under drought. *DFR, FGT2, PSI* and *PSII* were also increased in *SPL13*RNAi plants under drought stress.

We propose a model for a drought tolerance mechanism regulated by moderate levels of miR156 over-expression (Fig. 7g). The diagrammatic representation shows the central role of miR156 in regulating drought stress in alfalfa. MiR156 is induced by drought stress, which in turn silences *SPL13* [21]. Reduced expression of *SPL13* driven by miR156 and increased levels of *WD40–1* enhance *DFR* resulting in accumulation of anthocyanins. In moderate miR156OE plants, primary metabolites such as GABA, proline and sugars also accumulate for carbon-to-nitrogen balance and osmotic homeostasis. Induction of miR156 during drought stress also enhances phenotypic plasticity, such as longer roots and higher biomass to access more water from the rhizosphere. With reduced *SPL13* expression, miR156OE and *WD40–1*OE*,* higher photosynthesis efficiency is also achieved during drought stress. We conclude that moderate levels of miR156 expression silence *SPL13* and increase WD40–1 expression to fine-tune *DFR* expression for anthocyanin biosynthesis and regulate various developmental, physiological and biochemical processes in alfalfa leading to improved drought resilience.

## Methods

### Genetic material

#### miR156 overexpressing and *SPL13*RNAi plants

*Medicago sativa* L. N4.4.2 plants [87] were obtained from Dr. Daniel Brown (Agriculture and Agri-Food Canada) and used as wild-type genotypes. Plants over-expressing miR156 (miR156OE) at different levels (A8a, A8 and A11) and an empty vector control (EV) were generated previously in our laboratory and used in this experiment [13]. miR156 is slightly (0.5) elevated in A8a, but it is moderate (1.5) to higher (2.5) relative transcript level in A8 and A11, respectively [13]. The plants were grown in a fully automated greenhouse with 16-h light (380–450 W/m^2^), relative humidity (RH) of 70% and temperature of 25 ± 2 °C at the Agriculture and Agri-Food Canada London Research and Development Center, London, Ontario, Canada. Given that alfalfa is an obligatory outcross, we used vegetative cuttings for propagation according to Aung et al [13] to maintain genotypes throughout the study. Since miR156 down-regulates seven *SPL* genes (including *SPL13*) to regulate a network of downstream genes, we used *SPL13*RNAi genotypes (*SPL13*RNAi-2, *SPL13*RNAi-5 and *SPL13*RNAi-6) [21] selected for their low *SPL13* expression levels relative to wild-type alfalfa and other *SPL13*RNAi transgenic alfalfa plants.

#### Generation of WD40–1 overexpressing and WD40–1RNAi alfalfa plants

Four WD40–1OE (OE04, OE09, OE14 and OE15) and four WD40–1RNAi (R03, R04, R10 and R11) genotypes were generated to investigate the role of WD40–1 in drought tolerance. WD40–1 overexpression and downregulated genotypes were generated using constructs made from alfalfa homolog *WD40–1* (Medtr3g074070) using Gateway cloning system (Thermo Fisher Scientific, Mississauga ON). For overexpression studies, full-length *WD40–1* was amplified from alfalfa (*Medicago sativa*) cDNA using primers with AttB sites attached, forward (B1-WD40–1) and reverse (B2-WD40–1) (Additional file 1: Table S1) and cloned into the pDONR/Zeo entry vector. For downregulation studies, a 253 bp putative *WD40–1* fragment was amplified from alfalfa cDNA using AttB sites attached forward (B1-WD40–1-RNAi) and reverse (B2-WD40–1-RNAi) (Additional file 1: Table S1) primers and cloned into pDONR/Zeo entry vector.

After PCR screening and confirmation by sequencing, LR reactions were performed for the overexpression and RNAi constructs to recombine the fragments into the pMDC83 (overexpression) and pHELLSGATE12 (RNAi) vectors. Subsequently, overexpression and RNAi constructs were used to transform *Agrobacterium tumefaciens* strain EHA105 which was then used to transform alfalfa. QRT-PCR was then used to analyze WD40–1 gene expression in WD40–1-OE *WD40-1-*RNAi genotypes using primers WD1-qPCR-F and WD1-qPCR-R (Additional file 1: Table S1).

### Imposing drought stress

Drought stress was imposed on alfalfa plants devoid of water for 2 weeks at 30 days post vegetative propagation (juvenile vegetative stage) during which time plants were kept in a completely randomized design. Equal soil moisture levels were maintained before starting the experiment using a SM 100 soil moisture sensor (Spectrum Technologies Inc., Jakarta, Indonesia). At least four biological replicates were used per genotype per treatment for transcript and metabolite analysis, while 4 to 10 plants were used for physiological analysis (each replicate being an individual plant). The entire experiment was repeated under the same growth and drought stress conditions to test the repeatability of results. Leaves (newly developed upper leaves), stems (lower 5 cm internode close to soil) and roots (7.5 cm of main and auxiliary root tips) were harvested from miR156OE, *SPL13*RNAi, *WD40–1*OE, *WD40–1*RNAi, EV and wild-type plants depending on the experiment. Samples were flash frozen with liquid nitrogen and kept at − 80^0^ C for later metabolomic and transcriptomic analyses.

### Metabolite extraction for parallel LCMS and GCMS analysis

To explore miR156-related regulation of secondary metabolites and transient primary metabolites, extracts of stem, leaf and root tissues of drought-stressed miR156OE and control plants were subjected to Liquid Chromatography-Mass Spectrometry (LCMS) and Gas Chromatography-Mass Spectrometry (GCMS) analysis. Extraction of samples was performed according to Ayenew et al. [28] for parallel LCMS and GCMS analysis. Unless stated otherwise, chemicals used for the analysis were obtained from Sigma-Aldrich, Canada. Briefly, frozen 50 mg tissues were crushed with a RETCH-mill (Retsch Gmbh, 42,787 Haan, Germany) and stainless-steel beads. One milliliter prechilled extraction solution, methanol/chloroform/water (2.5/1/1 v/v/v), was added containing an internal standard Ribitol/adonitol 0.225 mg/mL for GCMS analysis while ampicillin (Sigma, and Saint Luis, Missouri, USA) and corticosterone at 1 mg/mL for LCMS to normalize extraction variability. The mixture was vortexed and ultra-sonicated for 10 min. Following centrifugation at 14000 rpm for 10 min (at 4^0^ C), supernatant was collected and mixed with equal volumes of 300 μL water and chloroform. The mixtures were vortexed briefly and centrifuged at 14000 rpm for 5 min to collect the upper aqueous phase for parallel LCMS and GCMS analyses.

LCMS analysis was performed using an Agilent 1290 Infinity LC system coupled with a Thermo Q-Exactive Quadrupole-Orbitrap mass spectrometer. Analytes were separated with an Agilent Eclipse Plus C18 ZORBAX Rapid Resolution High Definition (RRHD) 1.8 μm particle 2.1 i.d. X 50 mm column. The instrument was equipped with electrospray ionization (ESI) interface operating in a negative and positive ion mode for better metabolite identification. Metabolites were identified based on mass to charge ratio (*m/z*), retention time and fragmentation pattern in comparison to commercial standards, ChemSpider and ReSpect phytochemical databases [28, 71]. MZmine2 software [88] was also used for LCMS metabolite mass detection, chromatogram building, and the separation of overlapping peaks. In parallel, transient primary metabolites were explored using 75 μL aliquots of the extracted samples for LCMS using an Agilent 5975C Triple-Axis Detector MSD and 7890A GC system in splitless mode. The aliquots were dried using an Eppendorf Vacufuge™ concentrator (Hamburg, Germany), derivatized by 40 μL *O*-methylhydroxylamine hydrochloride in pyridine with 7 μL standard alkane mixture (0.029% v/v C10-C20 of each 50 mg/l) for 2 h at 37 °C followed by 70 μL *N*-methyl-*N*-[trimethylsilyl] trifluoroacetamide (MSTFA) for silylation. Metabolites from GCMS were identified using the retention time of the standard alkane mixture with their mass spectra and a NIST 2011 mass spectral library [27, 28, 72].

### Total monomeric anthocyanin and polyphenol determination

Total monomeric anthocyanin, TMA, and total polyphenol, TPP, were determined using a pH deferential extraction method [89, 90]. Briefly, flash-frozen in liquid nitrogen samples were crushed with mortar and pestle under liquid nitrogen and 500 mg tissue were used for the combined analysis of TMA and TPP. Samples were treated with 2 ml acidified methanol (MeOH with 1% HCL), vortexed and sonicated at 20 KHz for 15 min. Homogenate was stirred at 3000 RPM for 1 h and centrifuged (at 4 °C) for 10 min at full speed (14,000 RPM). The supernatant was collected, added 2 ml chloroform, vortexed and centrifuged at full speed for 10 min. The upper aqueous phase was collected, filtered with Whiteman 0.2 um filters, and divided into three equal aliquots for TMA (pH 1.0 and 4.5) and TPP analysis. The first aliquot was mixed with an equal volume of 0.025 M KCl at pH 1.0 while the second is mixed with equal volumes of 0.4 M sodium acetate at pH 4.5 and measured absorbance at 520 and 700 nm with water as a blank. TPP was analysed by mixing an equal volume of the third aliquot with Folin-chiocalteu reagent (diluted 1:10 with water) and vortexed for 3 min. Four ml of sodium carbonate (7.5% w/v) was added to the mixture, which was then vortexed and incubated for 30 min in the dark. TPP was determined as gallic acid equivalent (GAE) after measuring absorbance of the aliquot at 765 nm with acidified methanol as blank. TMA level is expressed as mg cyanidin-3-o-glucoside (CG) equivalent.

### Physiological and phenotypic data measurement

To determine drought mitigating strategies, we investigated phenotypic and physiological parameters. Midday photosynthesis assimilation rates and dark-adapted chlorophyll fluorescence (Fv/Fm) were measured in newly growing upper unshaded leaves using a LI-6400XT portable photosynthesis meter coupled with Fluorescence System (LI-COR Biosciences, Lincoln, Nebraska, USA). Photosynthetic assimilation rate responses across a gradient of CO_2_ level (A/Ci) in the mesophyll cells to determine the maximum rate of rubisco carboxylase activity (*Vcmax*) and maximum photosynthetic electron transport rate (*Jmax*) was calculated to determine photosynthetic efficiency using the R statistical software plantecophys package [91]. Chlorophyll concentration index (CCI) of newly growing upper leaves were also determined using an Apogee MC100 instrument (Apogee instruments, Logan, Utah, USA) [92]. To determine plant water status, the midday leaf water potential was measured using a SAPS II Portable Plant Water Status Console (Soilmoisture Equipment Corp., Santa Barbara, CA, USA) in dark-adapted leaves by covering leaves with a polyethylene bag and aluminium foil for 20 min. In addition, above and below ground phenotypic parameters were measured, such as stem number and shoot weight, root length and weight according to Aung et al. [13], and stem basal diameter at 1 cm above stem-soil interface.

### RNA extraction and qRT-PCR analysis

Stem, leaf and root samples were collected and flash frozen in liquid nitrogen and kept in a -80 °C freezer until further use. Approximately 50 mg fresh weight was used for total RNA extraction using a PowerPlant® RNA isolation kit (Cat # 13500) for leaf samples, a QIAGEN RNeasy® Plant mini kit for stem and root tissues (Cat # 74904), and a PowerLyzer®24 bench top bead-based homogenizer (Cat # 13155) following manufacturers protocols. The extracted RNA was treated with Ambion®TURBO DNA-*free™* DNase (Cat # AM1907) followed by iScript™ cDNA synthesis (Cat # 1708891).

Transcript levels of selected genes involved in secondary metabolite biosynthesis and photosynthesis were investigated in this study. Using publicly available transcriptomics data of two miR156OE alfalfa genotypes under control (unstressed) conditions [8] and *M. truncatula* genome sequence Mt4.0 V2 (http://www.medicagogenome.org/downloads), transcripts of differentially expressed genes with the SBD core GTAC sequence within 2.5 kb of their promoter regions were identified. Among those, genes shown by Gene Ontology analysis to be involved in flavonoid biosynthesis, photosynthetic efficiency and stress tolerance were chosen for expression analysis by qRT-PCR. Primers specific to the above genes (Additional file 1: Table S1) were designed using *M. truncatula* genome sequence and amplified product was sequenced for an identity check (Additional file 2: Figure S2). Publicly available Primer3 software (http://primer3.ut.ee/) was used to design primers, and their efficiency was verified at different concentrations with gradient annealing temperature PCR before using for qRT-PCR analysis.

QRT-PCR was performed using the CFX96™ Real-Time PCR detection system and SsoFast™ EvaGreen® Supermixes (Bio-Rad Cat # 1725204). Specifically, 2 μL cDNA (equivalent to 200 ng cDNA), 1 μL forward and reverse gene-specific primers (10 μM each), 5 μL SsoFast Eva green Supermix, and 2 μL of nuclease-free water was used to make the final reaction volume of 10 μL. PCR amplification was performed at: cDNA denaturation at 95 °C for 30 s followed by 40 cycles of 95 °C for 10 s, 58 °C for 30 s and 72 °C for 30 s (denaturation, annealing and extension, respectively) followed by a melting curve that runs from 65 °C to 95 °C with a gradual increment of 0.5 per 5 s. All reactions were performed with three technical and four biological replicates. Transcript levels were analysed relative to acetyl-CoA carboxylase (*ACC1*) and *ACTIN* housekeeping genes designed based on alfalfa sequence [13, 21].

### ChIP-qPCR analysis of SPL13-DNA binding

Shoot tips of alfalfa plants overexpressing *SPL13* tagged with GFP driven by the CaMV35S promoter (p35S:SPL13-GFP) [52] were used to understand the occupancy of SPL13 on promoters of downstream genes contributing to drought tolerance. One-month-old SPL13-GFP overexpressing genotypes and WT control plants were used for ChIP-qPCR analysis based on previously published protocol [93] with some modifications. Briefly, 500 mg of shoot tips from WT and p35S:SPL13-GFP plants were collected, washed, proteins bound to DNA were cross-linked using 1% formaldehyde and mixtures were ground with liquid nitrogen. Extraction reagents and buffers are listed in Additional file 2: Table S4. Powdered tissues were homogenized with 15 ml of prechilled Extraction Buffer 1 and filtered with two layers of Miracloth (Millipore, Canada). Subsequently, the filtered mixture was centrifuged at 3000 *g* for 20 min and supernatant was discarded while the pellets were resuspended in 1 ml of prechilled Extraction Buffer 2 and centrifuged at 12000 *g* for 10 min. Afterwards, pellets were resuspended in 300 μL prechilled Extraction Buffer 3 and centrifuged at 16000 *g* for 1 h. The supernatant was removed, and chromatin pellets were resuspended in 300 μL of Nuclei Lysis Buffer by gentle pipetting and sheared twice at power 3 for 15 s on ice using a Sonic Dismembrator (Fisher Scientific, USA). Twenty microliter of supernatant aliquots were kept aside for later use as an input DNA control while using the remaining solution for immunoprecipitation. Chromatin solution was brought to 1.5 mL using a ChIP dilution buffer and divided into two equal parts for chromatin immunoprecipitation and a negative control. To each tube, 30 μL of protein A-agarose beads (Millipore, Canada) were added and the mixture was gently agitated, centrifuged (3500 *g*) for 1 min, and supernatant was transferred for immunoprecipitation while discarding the beads. Five μL of Ab290 GFP antibody was added to one of the chromatin solutions (keeping the second one as a no-antibody negative control) for an overnight gentle agitation at 4 °C. After 12 h, 40 μL of protein A-agarose beads were added and immune complexes were recovered by centrifugation and washed with cycle of low normality salt, high salt, LiCl and TE buffer. Immunocomplexes were eluted from beads using 250 μL of Elution Buffer and cross linking was reversed with 20 μL of 5 M NaCl incubated at 65^0^ C for 5 h. To each sample 10 μL 0.5 M EDTA, 20 μL 1 M Tris-HCl (pH 6.5) and 2 μL of 10 mg/mL proteinase K (Sigma-Aldrich, Canada) were added. DNA was extracted using phenol: chloroform (1:1, v:v), recovered by precipitation with ethanol and 0.3 M sodium acetate (pH = 5.2) and 2 μL glycogen carrier 10 mg/mL (Sigma-Aldrich, Canada) after overnight incubation at -20 °C. After 12 h, the solution was centrifuged at full speed for 20 min to pellet the DNA and pellet was then washed with 70% ethanol, resuspended with 16 μL of distilled water, and DNA was used for ChIP-qPCR analysis. To obtain the *DFR* promoter region sequence from *M. sativa*, proDFR1-MTR primers (Additional file 1: Table S1) were designed using a close relative *M. truncatula* sequence and amplified region was cloned into TOP10 competent *E. coli* cells using CloneJET (Thermo Scientific) and sequenced. Subsequently, proDFR ChIP-qPCR primers (Additional file 1: Table S1) were designed based on alfalfa sequences. QRT-PCR was performed using ChIP-precipitated DNA as described above while fold enrichment was calculated by dividing Ct values of p35S:SPL13-GFP to WT and comparing with *LOB1* reference gene [52].

### Genome walking for WD40–1 promoter nucleotide sequence

Due to lack of alfalfa genome sequence, we used Clonetech GenomeWalker™ (California, USA Cat No. 638904) to obtain nucleotide sequence of the *WD40–1* promoter region. In brief, we extracted genomic DNA from wild-type alfalfa plants using a Nucleospin®Tissue DNA extraction kit (MACHEREY-NAGEL Gmbh & Co. KG Germany, Cat. No. 740952). GenomeWalker “libraries” were prepared by digesting the DNA with four different restriction enzymes (*Dra*I, *Eco*RV, *Pvu*II and *Stu*I) at 37 °C for 2 h to generate blunt ends. Subsequently, two nested PCR amplifications were performed sequentially for each library using gene specific primers (GSP1 and GSP2) and adapter primers (AP1 and AP2) from the kit (Additional file 1: Table S1). PCR products were analyzed on a 1.5% agarose gel followed by cloning into a pJET1.2 cloning vector to facilitate sequencing. Subsequently, sequences obtained from the four libraries were aligned together to generate the consensus promoter region sequence of WD40–1 in alfalfa.

### Statistical data analysis

Shapiro-Wilk test were used for checking the normal distribution of data before proceeding to analysis of variance (ANOVA). Subsequently, Tukey post hoc multiple comparison were done on molecular (qRT-PCR and ChIP-qPCR), metabolomics (LCMS and GCMS), physiological and phenotypic data. Pair-wise t-test comparison was implemented between WD40–1OE and wild-type plants and with WD40–1RNAi plants for WD40–1 transcript abundance. Metabolite profile data were subjected to pareto scaling before principal component analysis (PCA) in which metabolites were mean-centred followed by dividing with the square root of the standard deviation. All statistical data analyses were undertaken using R-software environment 3.2.5.

## Supplementary information

**Additional file 1:** **Table S1.** List of primers used and their nucleotide sequences.

**Additional file 2:** **Table S2.** LCMS-based metabolite profiles of drought stressed alfalfa plants. **Table S3** GCMS-based relative metabolite abundance in drought stressed alfalfa plants. **Table S4** Buffers used in ChIP assay and their components. **Table S5.1** Analysis of variance, ANOVA, *P* values of data for phenotype and physiological responses in miR156OE genotypes and EV plants. **Table S5.2** Analysis of variance, ANOVA, P values of data for phenotype, physiological and metabolite responses in SPL13RNAi genotypes and EV plants. **Table S5.3** Analysis of variance, ANOVA, P values of data for phenotype and physiological responses in WD40–1OE, WD40–1RNAi and wild type plants. **Table S5.4** Analysis of variance, ANOVA, P values of data for LCMS-based metabolite profiling in miR156OE genotypes and EV alfalfa plants. **Table S5.5** Analysis of variance, ANOVA, P values (P > F) of data for GCMS-based metabolite profiling in miR156OE genotypes and EV alfalfa plants. **Table S5.6** Analysis of variance, ANOVA, P values of data for qRT-PCR based transcript level in miR156OE genotypes and EV alfalfa plants. **Table S5.7** Analysis of variance, ANOVA, P values of data for qRT-PCR based transcript level in SPL13RNAi genotypes and EV alfalfa plants. **Table S5.8** Analysis of variance, ANOVA, P values of data for ChIP-qPCR based transcript level in p35S:SPL13-GFP genotypes and Wild-type alfalfa plants. **Table S5.9** Analysis of variance, ANOVA, P values of data for qRT-PCR based transcript level in WD40–1RNAi silenced and WD40–1over expressing plants. **Figure S1** Stem colour development in miR156OE plants during drought stress. **Figure S2** Alignment of sequences of amplified by q-PCR from *Medicago sativa* with those of their counterparts in *Medicago truncatula.* **Figure S3** Promoter sequence of the alfalfa *DIHYDROFLAVONOL-4-REDUCTASE (DFR)* gene with putative SBD binding elements. **Figure S4** Nucleotide sequence of the alfalfa *WD40–1* promoter region.

## Data Availability

Data used in this study are provided as ‘additional file.xlsx’ as a supplementary file.

## Abbreviations

ABA: Abscisic acid
DAG: Delphinidin 3-O-(6″-acetyl)-glucoside
*DFR*: *DIHYDROFLAVONOL-4-REDUCTASE*
*DRR*: *DEHYDRATION RESPONSIVE RD-22-LIKE*
EV: Empty vector
*FGT2*: *FLAVONOID GLUCOSYLTRANSFERASE2*
GABA: Gamma-aminobutyric-acid
GCMS: Gas chrmotaography mass spectrometry
LCMS: Liquid chromatography mass spectrometry
*LOB1*: *LATERAL ORGAN BOUNDARIES-LIKE1*
miR156: microRNA156
PA: Proanthocyanidins
*PAL*: *PHENYLALANINE AMMONIA-LYASE*
*PAP1*: *PRODUCTION OF ANTHOCYANIN PIGMENT 1*
PCA: Principal Component Analysis
PG: Peonidin 3-O-glucoside
PPP: Pentose phosphate pathway
*PSI*: *PHOTOSYSTEM I p700 CHLOROPHYLL A APOPROTEIN APS I* gene
PSII: Photosystem II
*PSII*: *PHOTOSYSTEM II Q(b)*gene
ROS: Reactive oxygen species
SBD: SPL binding domain
SPL: SQUAMOSA-PROMOTER BINDING PROTEIN-LIKE
WT: Wild-type
