# Supplementary material for: The interplay between miR156/SPL13 and DFR/WD40–1 regulate drought tolerance in alfalfa
Source: BMC Plant Biol. 2019 Oct 21;19:434. doi: 10.1186/s12870-019-2059-5 (PMC6802326; doi:10.1186/s12870-019-2059-5)

| **Name of compound** | **Formula** | **mz** | **RT** | **EV** | | | **A8a** | | | **A8** | | | **A11** | | |
| --- | --- | --- | --- | --- | --- | --- | --- | --- | --- | --- | --- | --- | --- | --- | --- |
|  |  |  |  | **Leaf** | **Stem** | **Root** | **Leaf** | **Stem** | **Root** | **Leaf** | **Stem** | **Root** | **Leaf** | **Stem** | **Root** |
| **Peonidin 3-O-glucoside** | C_22_H_23_O_11_ | 463.16 | 5.93 | 0.0044 | 0.0000 | 0.0342 | 0.0253 | 0.0330 | 0.0357 | 0.0189 | 0.0278 | 0.0282 | 0.0082 | 0.0300 | 0.0493 |
| **Delphinidin 3-O-(6''-acetyl)-glucoside** | C_23_H_23_O_13_ | 507.11 | 2.90 | 0.1069 | 0.0798 | 0.0012 | 0.1837 | 0.0612 | 0.0006 | 0.2452 | 0.0762 | 0.0002 | 0.1069 | 0.0454 | 0.0001 |
| **p-coumaric acid** | C_9_H_8_O_3_ | 163.02 | 1.95 | 0.0002 | 0.0018 | 0.0055 | 0.0007 | 0.0027 | 0.0064 | 0.0009 | 0.0026 | 0.0058 | 0.0005 | 0.0011 | 0.0082 |
| **Phenylalanine** | C_9_H_11_NO_2_ | 164.07 | 2.67 | 0.0309 | 0.0101 | 0.0100 | 0.0747 | 0.0355 | 0.0078 | 0.0907 | 0.0114 | 0.0540 | 0.0321 | 0.0075 | 0.0379 |
| **Ferulate** | C_10_H_10_O_4_ | 193.05 | 2.61 | 0.0010 | 0.0017 | 0.0072 | 0.0025 | 0.0019 | 0.0062 | 0.0039 | 0.0024 | 0.0050 | 0.0011 | 0.0014 | 0.0062 |
| **Astilbin** | C_21_H_22_O_11_ | 449.10 | 2.55 | 0.0030 | 0.0034 | 0.0678 | 0.0077 | 0.0032 | 0.0386 | 0.0095 | 0.0059 | 0.0358 | 0.0057 | 0.0034 | 0.0865 |
| **Quercetin** | C_15_H_10_O_7_ | 301.03 | 2.75 | 0.0031 | 0.0055 | 0.0004 | 0.0074 | 0.0043 | 0.0003 | 0.0092 | 0.0046 | 0.0003 | 0.0032 | 0.0048 | 0.0003 |
| **Tryptophan** | C_11_H_12_N_2_O_2_ | 203.08 | 2.76 | 0.0201 | 0.0113 | 0.0085 | 0.0454 | 0.0110 | 0.0140 | 0.0546 | 0.0169 | 0.0154 | 0.0109 | 0.0169 | 0.0146 |
| **Kaempferol 3-O-rutinose** | C_27_H_31_O_15_ | 595.17 | 2.44 | 0.0020 | 0.0013 | 0.0001 | 0.0025 | 0.0015 | 0.0001 | 0.0031 | 0.0016 | 0.0001 | 0.0013 | 0.0012 | 0.0000 |
| **trans-Cinnamate** | C_9_H_8_O_2_ | 148.05 | 2.37 | 0.0005 | 0.0022 | 0.0007 | 0.0012 | 0.0019 | 0.0006 | 0.0011 | 0.0020 | 0.0006 | 0.0003 | 0.0015 | 0.0007 |
| **Citric acid** | C_6_H_8_O_7_ | 191.02 | 0.52 | 0.0126 | 0.0237 | 0.0165 | 0.0294 | 0.0162 | 0.0063 | 0.0253 | 0.0130 | 0.0068 | 0.0108 | 0.0144 | 0.0220 |
| **Sucrose** | C_12_H_22_O_11_ | 341.12 | 0.52 | 0.0084 | 0.0199 | 0.0166 | 0.0461 | 0.0218 | 0.0204 | 0.0310 | 0.0363 | 0.0167 | 0.0492 | 0.0160 | 0.0247 |
| **4-hydroxybenzoic acid** | C_7_H_6_O_3_ | 139.04 | 2.19 | 0.0039 | 0.0102 | 0.0095 | 0.0142 | 0.0080 | 0.0082 | 0.0172 | 0.0087 | 0.0102 | 0.0051 | 0.0055 | 0.0089 |
| **caffeic acid** | C_9_H_8_O_4_ | 181.05 | 3.25 | 0.0032 | 0.0041 | 0.0064 | 0.0124 | 0.0041 | 0.0079 | 0.0130 | 0.0033 | 0.0060 | 0.0035 | 0.0018 | 0.0059 |
| **Catechin** | C_15_H_14_O_6_ | 291.10 | 2.33 | 0.0037 | 0.0083 | 0.0002 | 0.0104 | 0.0072 | 0.0003 | 0.0115 | 0.0064 | 0.0001 | 0.0029 | 0.0037 | 0.0000 |
| **Epicatechin** | C_15_H_14_O_6_ | 291.09 | 2.86 | 0.0086 | 0.0154 | 0.0135 | 0.0376 | 0.0086 | 0.0084 | 0.0454 | 0.0078 | 0.0102 | 0.0280 | 0.0106 | 0.0078 |
| **Corticosterone** | C_19_H_25_NO_5_ | 347.221 | 3.764 |  |  |  |  |  |  |  |  |  |  |  |  |
| **Ampicillin** | C_16_H_19_N_3_O_4_S | 350.117 | 2.433 |  |  |  |  |  |  |  |  |  |  |  |  |

**Table S2: LCMS-based metabolite profiles of drought stressed alfalfa plants**

Relative metabolite abundance values (peak area) are normalized to internal standard corticosterone and ampicillin. N=4 biological replicates from different plants obtained from drought exposed plants.

**Table S3. GCMS-based relative metabolite abundance in drought stressed alfalfa plants**

| **Metabolite** | **EV** | | | **A8a** | | | **A8** | | | **A11** | | |
| --- | --- | --- | --- | --- | --- | --- | --- | --- | --- | --- | --- | --- |
|  | **Leaf** | **Stem** | **Root** | **Leaf** | **Stem** | **Root** | **Leaf** | **Stem** | **Root** | **Leaf** | **Stem** | **Root** |
| **Alanine** | 0.08 | 0.13 | 0.11 | 0.04 | 1.69 | 1.80 | 0.32 | 0.69 | 0.60 | 0.07 | 0.12 | 0.28 |
| **Asparagine** | 0.83 | 2.32 | 2.38 | 0.96 | 0.73 | 2.42 | 2.02 | 0.98 | 2.51 | 0.56 | 0.44 | 0.23 |
| **Aspartate** | 1.97 | 2.15 | 2.18 | 1.31 | 1.41 | 2.34 | 1.83 | 2.50 | 3.27 | 1.41 | 2.86 | 1.98 |
| **Glycine** | 0.88 | 1.04 | 1.21 | 0.89 | 1.82 | 1.62 | 1.62 | 1.60 | 1.62 | 0.44 | 0.77 | 1.25 |
| **Hydroxylamine** | 0.79 | 1.65 | 1.48 | 0.56 | 1.31 | 1.92 | 1.06 | 1.83 | 2.05 | 1.35 | 1.97 | 1.67 |
| **Isoleucine** | 0.77 | 0.31 | 0.31 | 0.38 | 0.20 | 0.24 | 0.34 | 0.39 | 0.71 | 1.03 | 0.22 | 0.08 |
| **Proline** | 11.28 | 23.64 | 62.42 | 17.00 | 13.98 | 79.01 | 3.53 | 13.11 | 68.80 | 2.67 | 3.43 | 9.72 |
| **Serine** | 0.89 | 2.18 | 1.86 | 1.12 | 0.70 | 2.15 | 0.42 | 1.37 | 2.40 | 0.15 | 0.70 | 1.00 |
| **Threonine** | 0.12 | 0.16 | 0.47 | 0.74 | 0.73 | 0.57 | 0.35 | 0.41 | 0.40 | 0.08 | 0.07 | 0.34 |
| **Tryptophan** | 0.77 | 0.29 | 0.10 | 0.94 | 0.30 | 0.07 | 1.31 | 0.25 | 0.11 | 0.45 | 0.16 | 0.03 |
| **Val** | 0.43 | 0.65 | 0.82 | 0.51 | 0.36 | 0.77 | 0.66 | 1.06 | 0.55 | 0.14 | 0.21 | 0.39 |
| **Citrate** | 6.07 | 8.88 | 23.26 | 9.26 | 10.19 | 12.31 | 12.92 | 12.98 | 22.17 | 12.56 | 22.51 | 22.83 |
| **Fumarate** | 1.82 | 1.76 | 1.20 | 1.64 | 1.52 | 1.36 | 1.96 | 2.48 | 1.26 | 1.29 | 2.00 | 1.99 |
| **Malate** | 81.77 | 126.63 | 62.02 | 101.67 | 76.36 | 58.52 | 100.98 | 149.79 | 62.50 | 35.15 | 80.79 | 63.33 |
| **Succinate** | 1.94 | 2.47 | 2.64 | 1.78 | 2.89 | 2.73 | 5.60 | 5.20 | 2.80 | 1.40 | 1.61 | 3.33 |
| **GABA** | 0.78 | 3.04 | 5.45 | 1.72 | 1.91 | 8.86 | 2.08 | 2.05 | 9.12 | 0.38 | 0.36 | 2.96 |
| **Lactate** | 0.27 | 0.49 | 0.75 | 0.64 | 0.49 | 1.41 | 0.73 | 1.06 | 1.37 | 0.55 | 0.67 | 1.19 |
| **Xylitol** | 1.82 | 0.69 | 1.04 | 0.55 | 0.33 | 1.67 | 2.57 | 2.25 | 1.36 | 0.99 | 0.53 | 0.35 |
| **Xylulose** | 38.14 | 6.30 | 11.84 | 49.87 | 3.30 | 12.40 | 38.74 | 6.56 | 13.58 | 14.54 | 3.27 | 0.42 |
| **Sucrose** | 436.64 | 626.02 | 675.26 | 350.48 | 439.24 | 940.36 | 436.28 | 571.35 | 683.04 | 256.67 | 431.57 | 858.28 |
| **Ribose** | 1.25 | 0.99 | 1.20 | 1.56 | 0.66 | 1.69 | 1.37 | 1.08 | 1.98 | 0.71 | 0.64 | 1.36 |
| **Fructose** | 5.93 | 49.79 | 12.65 | 17.70 | 40.28 | 10.71 | 23.40 | 53.43 | 15.45 | 10.61 | 13.04 | 21.42 |
| **Arabinose** | 2.04 | 0.63 | 1.77 | 1.46 | 2.16 | 2.38 | 1.44 | 2.02 | 2.29 | 0.71 | 0.64 | 1.51 |
| **Allose** | 67.68 | 259.53 | 48.54 | 57.02 | 224.51 | 104.09 | 131.58 | 294.98 | 152.09 | 44.07 | 200.93 | 214.20 |
| **Aconitate** | 1.83 | 1.58 | 0.51 | 1.05 | 0.77 | 0.37 | 1.74 | 1.10 | 0.66 | 1.75 | 1.36 | 0.38 |
| **Azelaiate** | 0.17 | 0.11 | 0.07 | 0.46 | 0.25 | 0.12 | 0.09 | 0.27 | 0.14 | 0.07 | 0.11 | 0.06 |
| **Benzoate** | 1.54 | 0.49 | 0.40 | 0.80 | 1.10 | 0.67 | 0.89 | 0.94 | 0.78 | 0.56 | 0.72 | 0.57 |
| **Butan** | 0.39 | 0.63 | 0.21 | 0.37 | 0.07 | 0.95 | 1.29 | 1.92 | 0.84 | 0.10 | 0.21 | 0.11 |
| **Callobiose** | 5.71 | 0.43 | 0.29 | 0.47 | 0.19 | 0.41 | 6.69 | 0.29 | 1.26 | 2.12 | 0.16 | 0.18 |
| **Erythrose** | 1.50 | 0.68 | 0.77 | 1.88 | 0.73 | 0.79 | 1.68 | 0.72 | 1.02 | 0.66 | 0.37 | 0.36 |
| **Glucarate** | 0.28 | 0.13 | 0.06 | 0.24 | 0.26 | 0.07 | 0.21 | 0.14 | 0.08 | 0.15 | 0.10 | 0.14 |
| **Gluconate** | 2.87 | 3.05 | 0.86 | 2.80 | 1.98 | 1.48 | 2.96 | 3.26 | 1.66 | 1.49 | 2.10 | 1.09 |
| **Glutarate** | 0.11 | 0.12 | 0.14 | 0.08 | 0.08 | 0.21 | 0.08 | 0.14 | 0.17 | 0.09 | 0.12 | 0.23 |
| **Glycerate** | 0.18 | 0.16 | 0.20 | 0.53 | 0.31 | 0.32 | 0.31 | 0.27 | 0.34 | 0.09 | 0.11 | 0.22 |
| **Inositol** | 66.88 | 10.45 | 8.40 | 120.06 | 13.06 | 9.75 | 70.45 | 11.87 | 9.34 | 28.04 | 13.13 | 7.11 |
| **Itaconate** | 0.07 | 0.29 | 0.27 | 0.24 | 0.27 | 0.36 | 0.14 | 0.36 | 0.29 | 0.31 | 0.40 | 0.30 |
| **Maleate** | 1.36 | 1.39 | 0.95 | 1.28 | 2.98 | 1.27 | 1.85 | 1.44 | 1.06 | 1.78 | 1.49 | 1.73 |
| **Malonate** | 12.56 | 37.82 | 14.41 | 6.03 | 11.63 | 17.04 | 16.33 | 28.08 | 14.77 | 5.72 | 14.76 | 9.27 |
| **Maltose** | 0.11 | 1.52 | 0.52 | 0.21 | 1.35 | 1.20 | 0.75 | 0.89 | 1.21 | 1.22 | 1.15 | 1.38 |
| **Mannitol** | 1.65 | 13.33 | 5.49 | 2.14 | 8.17 | 10.92 | 2.48 | 15.17 | 13.96 | 1.81 | 6.34 | 21.12 |
| **Phosphoric acid** | 1.42 | 39.58 | 53.00 | 1.77 | 27.39 | 63.53 | 1.55 | 48.25 | 88.46 | 0.90 | 52.35 | 38.28 |
| **PineridineC** | 0.29 | 0.39 | 0.62 | 0.29 | 0.36 | 0.82 | 0.67 | 0.24 | 0.68 | 0.21 | 0.35 | 0.09 |
| **Pinitol** | 548.49 | 558.43 | 485.53 | 611.33 | 511.23 | 567.52 | 591.56 | 678.14 | 594.62 | 249.64 | 369.53 | 395.88 |
| **Pinitol-2** | 63.03 | 21.86 | 21.26 | 55.04 | 18.52 | 19.30 | 64.81 | 23.92 | 19.36 | 19.59 | 18.37 | 14.80 |
| **Tartarate** | 0.78 | 1.18 | 1.14 | 0.39 | 0.43 | 1.61 | 0.50 | 0.82 | 1.29 | 0.27 | 0.79 | 0.50 |
| **Threonate** | 2.52 | 2.55 | 0.43 | 2.60 | 1.59 | 0.54 | 4.08 | 2.61 | 0.47 | 1.57 | 2.17 | 0.43 |
| **Turanose** | 4.61 | 5.64 | 0.07 | 1.98 | 3.35 | 0.11 | 4.23 | 4.29 | 0.12 | 0.81 | 2.02 | 0.17 |
| **1279NA** | 0.19 | 0.15 | 0.18 | 0.31 | 0.14 | 0.10 | 0.16 | 0.41 | 0.19 | 0.07 | 0.17 | 0.16 |

Relative metabolite abundance values (peak area) are normalized to internal standard corticosterone and ampicillin. N=4 biological replicates from different plants obtained from drought exposed plants. 1279NA is unidentified metabolite using in-house metabolite database.

**Table S4:** **Buffers used in ChIP assay and their components**

| Buffers | Chemicals | Concentration | Buffers | Chemicals | Concentration |
| --- | --- | --- | --- | --- | --- |
| Extraction buffer 1 | Sucrose | 0.4 M | Extraction buffer 2 | Sucrose | 0.25 M |
|  | Tris-HCl (pH=8) | 10 mM |  | Tris-HCl (pH=8) | 10 mM |
|  | MgCl_2_ | 10 mM |  | MgCl_2_ | 10 mM |
|  | β-ME | 5 mM |  | Triton X-100 | 1% |
|  | PMSF | 0.1 mM |  | β-ME | 5 mM |
|  | Protease inhibitor^1^ | 2 tablets/ 100mL |  | PMSF | 0.1 mM |
|  |  |  |  | Protease inhibitor^1^ | 1 tablet/10mL |
| Extraction buffer 3 | Sucrose | 1.7 M | Nuclei lysis buffer | Tris-HCl (pH=8) | 50 mM |
|  | Tris-HCl (pH=8) | 10 mM |  | EDTA | 10 mM |
|  | MgCl_2_ | 2 mM |  | SDS | 1% |
|  | Triton X-100 | 0.15% |  | Protease inhibitor^1^ | 1 tablet/10mL |
|  | β-ME | 5 mM | ChIP dilution buffer | Triton X-100 | 1.10% |
|  | PMSF | 0.1 mM |  | EDTA | 1.2 mM |
|  | Protease inhibitor^1^ | 1 tablet/10mL |  | Tris-HCl (pH=8) | 16.7 mM |
|  | Sucrose | 1.7 M |  | NaCl | 167 mM |
| Elution buffer | SDS | 1% | High salt wash buffer | SDS | 0.10% |
|  | NaHCO_3_ | 0.1M |  | Triton X-100 | 1% |
| Low salt wash buffer | SDS | 0.10% |  | EDTA | 2 mM |
|  | Triton X-100 | 1% |  | Tris-HCl pH=8) | 20 mM |
|  | EDTA | 2 mM |  | NaCl | 500 mM |
|  | Tris-HCl (pH=8) | 20 mM | LiCl wash buffer | LiCl | 0.25 M |
|  | NaCl | 150 mM |  | IGEPAL-CA630 | 1% |
|  |  |  |  | Deoxycholic acid | 1% |
| TE buffer | EDTA | 1 mM |  | EDTA | 1 mM |
|  | Tris-HCl (pH=8) | 10 mM |  | Tris-HCl (pH=8) | 10 mM |

^1^ Obtained from Sigma-Aldrich, Canada

| **Table S5.1** Analysis of variance, ANOVA, P values of data for phenotype and physiological responses in miR156OE genotypes and EV plants | | | | | | | | | | | | | | | | | |  | | | | | |  | | | |  | | | |  | | | |  | | | |  | | | |  |  |  |
| --- | --- | --- | --- | --- | --- | --- | --- | --- | --- | --- | --- | --- | --- | --- | --- | --- | --- | --- | --- | --- | --- | --- | --- | --- | --- | --- | --- | --- | --- | --- | --- | --- | --- | --- | --- | --- | --- | --- | --- | --- | --- | --- | --- | --- | --- | --- |
| Physiological parameter | Genotype | | Exposure time | Trt | Genotype* time | Genotype*Trt | | Trt*Time | | | |  | Genotype*time*trt | | | |  | |  | | | | | |  | | | |  | | | |  | | | |  | | | |  | | | |  |  |
| Root weight | 0.0348 | | - | - | - |  | - |  | |  | | |  | |  | | | | |  | |  | | | |  | | | |  | | | |  | | | |  | | | |  | | | |  |
| Root length | 0.044 | | - | - | - |  | - |  | |  | | |  | |  | | | | |  | |  | | | |  | | | |  | | | |  | | | |  | | | |  | | | |  |
| Stem basal width change | 0.0216 | | - | - | - |  | - |  | |  | | |  | |  | | | | |  | |  | | | |  | | | |  | | | |  | | | |  | | | |  | | | |  |
| Root to shoot ratio | 0.0376 | | - | NS | - |  | NS |  | |  | | |  | |  | | | | |  | |  | | | |  | | | |  | | | |  | | | |  | | | |  | | | |  |
| Leaf water potential | 0.037427 | | - | 1.17E-10 | - |  | 0.000449 |  | |  | | |  | |  | | | | |  | |  | | | |  | | | |  | | | |  | | | |  | | | |  | | | |  |
| Vc max | 1.85E-06 | | - | 5.98E-15 | - |  | 1.30E-09 |  | |  | | |  | |  | | | | |  | |  | | | |  | | | |  | | | |  | | | |  | | | |  | | | |  |
| Jmax | 2.60E-09 | | - | 2.00E-16 | - |  | 1.48E-12 |  | |  | | |  | |  | | | | |  | |  | | | |  | | | |  | | | |  | | | |  | | | |  | | | |  |
| Fv/FM | 2.00E-16 | | 2.00E-16 | 2.00E-16 | 7.70E-13 |  | 2.00E-16 |  | | 2.00E-16 | | |  | | 5.74E-13 | | | | |  | |  | | | |  | | | |  | | | |  | | | |  | | | |  | | | |  |
| Stomatal conductance | 2.82E-06 | | NS | 1.32E-02 | 3.10E-02 |  | 9.10E-03 |  | | 0.00124 | | |  | | 1.51E-07 | | | | |  | |  | | | |  | | | |  | | | |  | | | |  | | | |  | | | |  |
| Photosynthetic assimilation rate | 1.57E-07 | | 2.00E-16 | 2.00E-16 | 7.29E-03 |  | 0.00441 |  | | 2.00E-16 | | |  | | 0.01459 | | | | |  | |  | | | |  | | | |  | | | |  | | | |  | | | |  | | | |  |
| NS=not significant at 95 % confidence interval | | |  |  |  |  |  |  | |  | | |  | |  | | | | |  | |  | | | |  | | | |  | | | |  | | | |  | | | |  | | | |  |
|  |  | |  |  |  |  |  |  | |  | | |  | |  | | | | |  | |  | | | |  | | | |  | | | |  | | | |  | | | |  | | | |  |
| **Table S5.2** Analysis of variance, ANOVA, P values of data for phenotype, physiological and metabolite responses in SPL13RNAi genotypes and EV plants | | | | | | | | | | | | | | | | | | | | | | | | | | | |  | | | |  | | | |  | | | |  | | | |  |  |  |
| Physiological parameter | | Genotype | Exposure time | Trt | Genotype* time | Genotype*Trt | | |  | |  | | |  | |  | | | | |  | |  | | | |  | | | |  | | | |  | | | |  | | | |  | | | |
| Leaf water potential | | 0.0331 | - | - | - |  | - |  | |  | | |  | |  | | | | |  | |  | | | |  | | | |  | | | |  | | | |  | | | |  | | | |  |
| Fv/FM | | 0.01192 | 7.93E-07 | - | 0.000729 |  | - |  | |  | | |  | |  | | | | |  | |  | | | |  | | | |  | | | |  | | | |  | | | |  | | | |  |
| Total monomeric anthocyanin, TMA | | 1.17E-06 | - | 0.000627 | - |  | NS |  | |  | | |  | |  | | | | |  | |  | | | |  | | | |  | | | |  | | | |  | | | |  | | | |  |
| Total polyphenol, TPP | | NS | - | NS | - |  | NS |  | |  | | |  | |  | | | | |  | |  | | | |  | | | |  | | | |  | | | |  | | | |  | | | |  |
| NS=not significant at 95 % confidence interval | | |  |  |  |  |  |  | |  | | |  | |  | | | | |  | |  | | | |  | | | |  | | | |  | | | |  | | | |  | | | |  |
|  |  | |  |  |  |  |  |  | |  | | |  | |  | | | | |  | |  | | | |  | | | |  | | | |  | | | |  | | | |  | | | |  |
| **Table S5.3** Analysis of variance, ANOVA, P values of data for phenotype and physiological responses in WD40-1OE, WD40-1RNAi and wild type plants | | | | | | | | | | | | | | | | | | | | | | | | | | | | | | | | | | | | | | | |  | | | |  |  |  |
| Physiological parameter | Genotype | | Trt | Genotype*Trt | |  |  |  | |  | | |  | |  | | | | |  | |  | | | |  | | | |  | | | |  | | | |  | | | |  | | | |  |
| Chlorophyll concentration | 2.00E-16 | | 2.00E-16 | 2.00E-16 |  |  |  |  | |  | | |  | |  | | | | |  | |  | | | |  | | | |  | | | |  | | | |  | | | |  | | | |  |
| Root length | 1.59E-15 | | NS | 0.000266 |  |  |  |  | |  | | |  | |  | | | | |  | |  | | | |  | | | |  | | | |  | | | |  | | | |  | | | |  |
| Root weight | 8.51E-04 | | - | - |  |  |  |  | |  | | |  | |  | | | | |  | |  | | | |  | | | |  | | | |  | | | |  | | | |  | | | |  |
| Leaf water potential | 0.00172 | | 6.78E-05 | NS |  |  |  |  | |  | | |  | |  | | | | |  | |  | | | |  | | | |  | | | |  | | | |  | | | |  | | | |  |
| NS=not significant at 95 % confidence interval | | |  |  |  |  |  |  | |  | | |  | |  | | | | |  | |  | | | |  | | | |  | | | |  | | | |  | | | |  | | | |  |

| **Table S5.4** Analysis of variance, ANOVA, P values of data for LCMS-based metabolite profiling in miR156OE genotypes and EV alfalfa plants | | | | | | | |
| --- | --- | --- | --- | --- | --- | --- | --- |
| Metabolite | | | Genotype | | Tissue | Genotype*Tissue | |
| Delphinidin3_O_6_acetyl_glucoside | | | 0.0489 | | 4.95E-10 | NS | |
| Peonidin3_Oglucoside | | | 0.03013 | | 0.000921 | NS | |
| Astilbin | | | 3.18E-05 | | 2E-16 | 1.39E-08 | |
| p_coumaricAcid | | | NS | | 2.66E-08 | NS | |
| Phenylanine | | | 0.00998 | | 0.000179 | 0.028045 | |
| Ferulate | | | NS | | 1.71E-10 | 0.0463 | |
| Quercetin | | | 0.013026 | | 7.90E-12 | 0.000152 | |
| Tryptophan | | | NS | | 0.00135 | NS | |
| Kaempferol3_O_rutinose | | | NS | | 2.11E-10 | NS | |
| trans_Cinnamate | | | NS | | 1.48E-09 | NS | |
| CitricAcid | | | NS | | 0.048284 | 0.000212 | |
| Sucrose | | | NS | | NS | NS | |
| 4_hydroxybenzoicAcid | | | 1.10E-05 | | NS | 1.31E-06 | |
| CaffeicAcid | | | 3.00E-06 | | 2.70E-07 | 4.82E-06 | |
| Catechin | | | 3.44E-08 | | 2.00E-16 | 1.12E-08 | |
| Epicatechin | | | NS | | 3.92E-06 | 0.00346 | |
| NS=not significant at 95 % confidence interval | | |  | |  |  | |
|  | | |  | |  |  | |
| **Table S5.5** Analysis of variance, ANOVA, P values (P>F) of data for GCMS-based metabolite profiling in miR156OE genotypes and EV alfalfa plants | | | | | | | |
| Metabolite | | | Genotype | | Tissue | Genotype*Tissue | |
| Alanine | | | 1.05E-06 | | 0.000821 | 0.001907 | |
| Aspartate | | | 0.000346 | | 1.27E-05 | 0.000836 | |
| Glycine | | | 7.19E-09 | | 1.98E-05 | 0.00312 | |
| Hydroxylamine | | | 5.98E-05 | | 1.88E-12 | 0.00151 | |
| Isoleucine | | | NS | | 0.00118 | 0.00143 | |
| Proline | | | 2.00E-16 | | 2.00E-16 | 2.96E-15 | |
| Serine | | | 1.32E-08 | | 4.53E-12 | 2.42E-06 | |
| threonine | | | 1.23E-12 | | 0.010143 | 0.000385 | |
| Threonine | | | 2.77E-05 | | 2.00E-16 | 0.000103 | |
| val | | | 7.51E-05 | | NS | 0.0176 | |
| Citrate | | | 2.32E-07 | | 3.31E-10 | 8.36E-05 | |
| Fumarate | | | NS | | NS | NS | |
| Malate | | | 6.29E-05 | | 9.86E-07 | 0.000364 | |
| Succinate | | | 2.64E-05 | | NS | 0.00571 | |
| GABA | | | 5.43E-15 | | 2.00E-16 | 4.85E-11 | |
| Lactate | | | 1.87E-05 | | 6.44E-09 | NS | |
| xylitol | | | 4.03E-07 | | 0.020056 | 0.000705 | |
| xylulose | | | 2.85E-06 | | 1.83E-15 | 0.000674 | |
| Sucrose | | | NS | | 1.79E-07 | NS | |
| Ribose | | | 1.52E-06 | | 2.16E-10 | 0.000455 | |
| Fructose | | | 0.006994 | | 1.91E-08 | 0.000374 | |
| Arabinose | | | 0.00332 | | 0.03029 | NS | |
| Allose | | | 0.000496 | | 9.08E-14 | 7.51E-05 | |
| Aconitate | | | 0.00137 | | 2.69E-10 | NS | |
| Azelaiate | | | 2.10E-06 | | 0.00103 | 8.17E-05 | |
| Benzoate | | | NS | | 0.010284 | 0.000781 | |
| Butan | | | 4.37E-09 | | NS | 0.000566 | |
| callobiose | | | 1.47E-12 | | 2.00E-16 | 4.06E-13 | |
| Erythrose | | | 4.08E-08 | | 1.83E-11 | 0.0169 | |
| Glucarate | | | 0.032559 | | 9.94E-09 | 0.000148 | |
| Gluconate | | | 0.00418 | | 1.52E-06 | NS | |
| Glutarate | | | NS | | 1.59E-09 | 0.00927 | |
| Glycerate | | | 9.65E-10 | | 0.03612 | 0.00222 | |
| Inositol | | | 1.13E-10 | | 2.00E-16 | 6.58E-13 | |
| Itaconate | | | 0.016425 | | 0.000302 | NS | |
| maleate | | | NS | | NS | NS | |
| malonate | | | 0.002007 | | 0.000266 | 0.04193 | |
| maltose | | | 0.0011 | | 7.96E-07 | 2.31E-05 | |
| mannitol | | | 0.000614 | | 7.79E-15 | 3.13E-09 | |
| phosphoric acid | | | 4.56E-06 | | 2.00E-16 | 1.10E-08 | |
| pineridineC | | | 0.0124 | | 0.0304 | 0.0142 | |
| pinitol | | | 7.47E-05 | | NS | NS | |
| pinitol-2 | | | 4.35E-08 | | 2.00E-16 | 3.46E-07 | |
| Tartarate | | | 0.000106 | | 3.56E-08 | 3.19E-05 | |
| threonate | | | 1.50E-05 | | 2.26E-16 | 2.44E-05 | |
| turanose | | | 6.04E-06 | | 1.13E-11 | 0.00465 | |
| 1279NA | | | NS | | NS | 0.0126 | |
| NS=not significant at 95 % confidence interval | | |  | |  |  | |
| **Table S5.6** Analysis of variance, ANOVA, P values of data for qRT-PCR based transcript level in miR156OE genotypes and EV alfalfa plants | | | | | | |  |
| Gene name | Genotype | Tissue | | Genotype*Tissue | | |  |
| *DFR* | 0.000136 | 7.16E-07 | | 0.013757 | | |  |
| *MYB112* | 1.76E-05 | 0.00035 | | 6.02E-05 | | |  |
| *WD40-1* | NS | 5.01E-05 | | 7.70E-03 | | |  |
| *FGT2* | 1.96E-11 | 6.55E-08 | | 3.39E-11 | | |  |
| *PSI* | NS | 2.03E-03 | | 0.03565 | | |  |
| *PSII* | 3.51E-07 | 2.62E-07 | | 2.71E-05 | | |  |
| NS=not significant at 95 % confidence interval | | | | | | |  |

|  |  |  |  |
| --- | --- | --- | --- |
| **Table S5.7** Analysis of variance, ANOVA, P values of data for qRT-PCR based transcript level in SPL13RNAi genotypes and EV alfalfa plants | | | |
| Gene name | Genotype |  |  |
| *PAL* | 0.000723 |  |  |
| *DFR* | 4.07E-06 |  |  |
| *MYB112* | NS |  |  |
| *WD40-1* | 0.000905 |  |  |
| *FGT2* | 8.16E-06 |  |  |
| *DRR* | 0.000922 |  |  |
| *PSI* | 1.63E-06 |  |  |
| *PSII* | 3.11E-08 |  |  |
| NS=not significant at 95 % confidence interval | | | |
|  |  |  |  |
| **Table S5.8** Analysis of variance, ANOVA, P values of data for ChIP-qPCR based transcript level in p35S:SPL13-GFP genotypes and Wild-type alfalfa plants | | | |
| Gene name | Genotype |  |  |
| *LOB1* | NS |  |  |
| *DFR1* | NS |  |  |
| *DFR2* | 0.0218 |  |  |
| *DFR3* | 0.0243 |  |  |
| NS=not significant at 95 % confidence interval | | | |
|  |  |  |  |
| **Table S5.9** Analysis of variance, ANOVA, P values of data for qRT-PCR based transcript level in WD40-1RNAi silenced and WD40-1over expressing plants | | | |
| Gene name | Genotype | Trt | Genotype*Trt |
| *PAL* | 5.22E-06 | 0.00069 | 7.03E-08 |
| *DFR* | 2.00E-16 | 2.00E-16 | 2.00E-16 |
| *FGT2* | 1.41E-07 | 8.55E-07 | 0.000276 |
| *DRR* | 8.95E-11 | 5.87E-08 | 1.90E-08 |
| *PSI* | 1.59E-09 | NS | NS |
| *PSII* | 1.82E-06 | NS | 0.000224 |
| NS=not significant at 95 % confidence interval | | | |

**Fig. S1 Stem colour development in miR156OE plants during drought stress.** Drought stressed EV plants and moR156OE plants on the on the left and right respectively


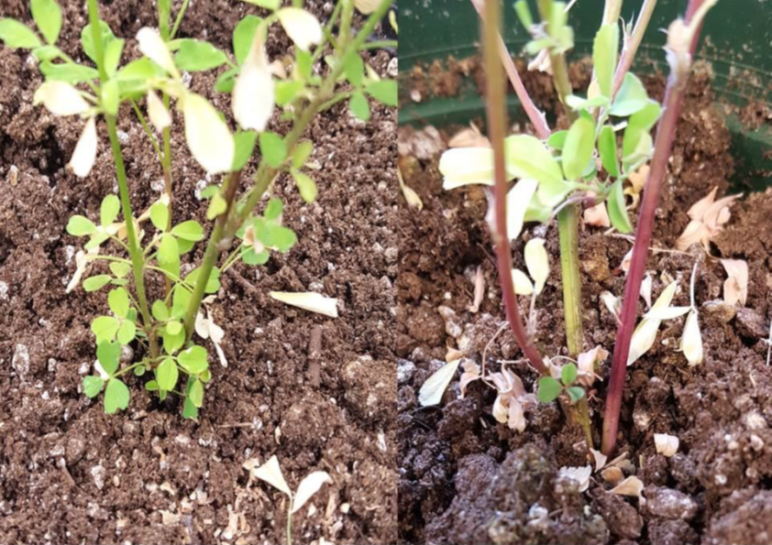


**Fig. S2:** **Alignment of sequences of amplified by q-PCR from *Medicago sativa* with those of their counterparts in *Medicago truncatula***. M. tr: Medicago truncatula nucleotide sequence and M. st: *Medicago sativa* nucleotide sequence followed by gene name: *DIHYDROFLAVONOL 4-REDUCTASE (DFR), MYB112, PHOTOSYSTEM I p700 CHLOROPHYLL A APOPROTEIN APS I (PSI), PHOTOSYSTEM II Q(b) (PSII), FLAVONOID GLUCOSYLTRANSFERASE2 (FGT2), PHENYLALANINE AMMONIA-LYASE (PAL), DEHYDRATION RESPONSIVE RD-22-LIKE (DRR)*


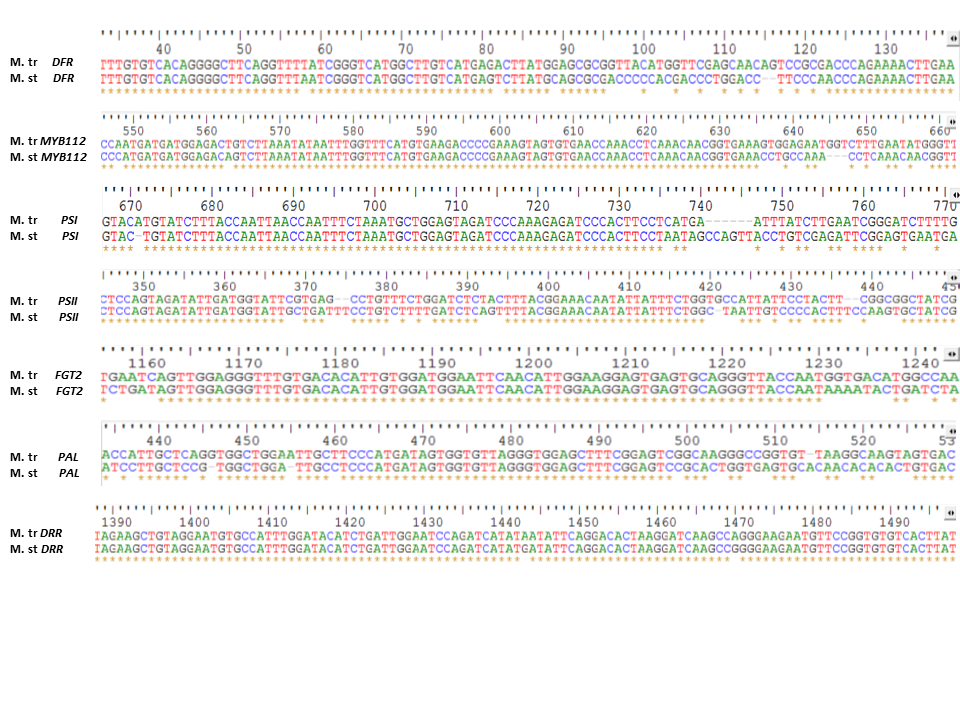


**Fig. S3: Promoter sequence of the alfalfa *DIHYDROFLAVONOL-4-REDUCTASE* *(DFR)* gene with putative SBD binding elements.** Nucleotides are highlighted with green, yellow and gray color represents putative SBD binding motifs with ‘GTAC’ core sequences, forward primer sequences used for ChIP-qPCR, and coding sequences of *DIHYDROFLAVONOL 4-REDUCTASE (DFR)* respectively.

**Fig. S4: Nucleotide sequence of the alfalfa *WD40-1* promoter region.** Nucleotides highlighted with gray color represent coding sequences of WD40-1 in Medicago sativa.


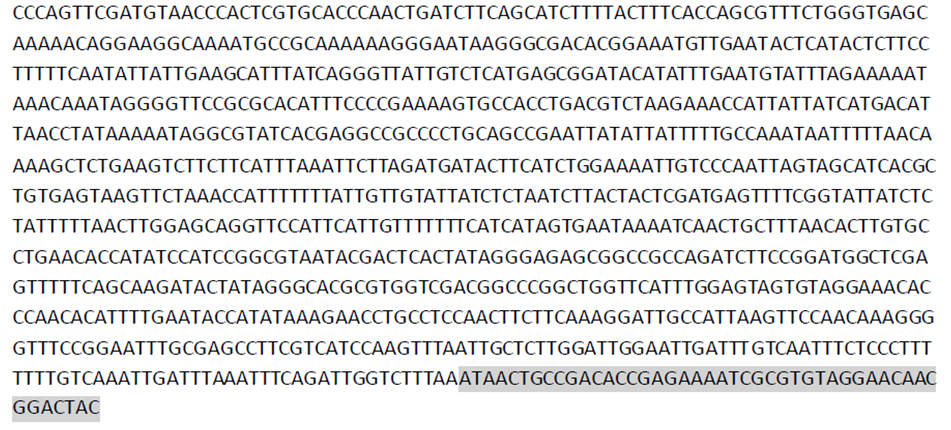

Supplement: Supplementary file 2 — Additional file 2: Table S2. LCMS-based metabolite profiles of drought stressed alfalfa plants. Table S3 GCMS-based relative metabolite abundance in drought stressed alfalfa plants. Table S4 Buffers used in ChIP assay and their components. Table S5.1 Analysis of variance, ANOVA, P values of data for phenotype and physiological responses in miR156OE genotypes and EV plants. Table S5.2 Analysis of variance, ANOVA, P values of data for phenotype, physiological and metabolite responses in SPL13RNAi genotypes and EV plants. Table S5.3 Analysis of variance, ANOVA, P values of data for phenotype and physiological responses in WD40–1OE, WD40–1RNAi and wild type plants. Table S5.4 Analysis of variance, ANOVA, P values of data for LCMS-based metabolite profiling in miR156OE genotypes and EV alfalfa plants. Table S5.5 Analysis of variance, ANOVA, P values (P > F) of data for GCMS-based metabolite profiling in miR156OE genotypes and EV alfalfa plants. Table S5.6 Analysis of variance, ANOVA, P values of data for qRT-PCR based transcript level in miR156OE genotypes and EV alfalfa plants. Table S5.7 Analysis of variance, ANOVA, P values of data for qRT-PCR based transcript level in SPL13RNAi genotypes and EV alfalfa plants. Table S5.8 Analysis of variance, ANOVA, P values of data for ChIP-qPCR based transcript level in p35S:SPL13-GFP genotypes and Wild-type alfalfa plants. Table S5.9 Analysis of variance, ANOVA, P values of data for qRT-PCR based transcript level in WD40–1RNAi silenced and WD40–1over expressing plants. Figure S1 Stem colour development in miR156OE plants during drought stress. Figure S2 Alignment of sequences of amplified by q-PCR from Medicago sativa with those of their counterparts in Medicago truncatula. Figure S3 Promoter sequence of the alfalfa DIHYDROFLAVONOL-4-REDUCTASE (DFR) gene with putative SBD binding elements. Figure S4 Nucleotide sequence of the alfalfa WD40–1 promoter region. [file 12870_2019_2059_MOESM2_ESM.docx]
